# Understanding ethnic inequalities in mental healthcare in the UK: A meta-ethnography

**DOI:** 10.1371/journal.pmed.1004139

**Published:** 2022-12-13

**Authors:** Narinder Bansal, Saffron Karlsen, Sashi P. Sashidharan, Rachel Cohen, Carolyn A. Chew-Graham, Alice Malpass

**Affiliations:** 1 Population Health Sciences, Bristol Medical School, University of Bristol, Bristol, United Kingdom; 2 Centre for the Study of Ethnicity and Citizenship, School of Sociology, Politics and International Studies, University of Bristol, Bristol, United Kingdom; 3 Institute of Health and Wellbeing, University of Glasgow, Glasgow, United Kingdom; 4 School of Medicine, Keele University, Keele, United Kingdom

## Abstract

**Background:**

Evidence regarding the presence and persistence of ethnic inequalities in mental healthcare is well established. The reasons for these inequalities and lack of progress in diminishing them are less understood. This meta-ethnography aims to provide a new conceptual understanding of how ethnic inequalities are created and sustained; this is essential to develop effective interventions. Specifically, we sought to understand why people from ethnic minority groups are underrepresented in primary care mental health service provision and overrepresented in crisis pathways and detention.

**Methods and findings:**

Following eMERGe guidelines for meta-ethnographies, we searched OpenGrey, Kings Fund, CINAHL, Medline, PsycINFO, and Social Care Online databases for qualitative articles published from database inception until October 2, 2022, using broad categories of search terms relating to “ethnicity AND (mental illness/mental health/emotional distress) AND (help-seeking/service utilisation/experience/perception/view).” We included all conceptually rich articles that used qualitative methods of data collection and analysis and excluded non-UK studies and those that focused solely on causation of mental illness. Our patient, public, and practitioner lived experience advisory group provided feedback and input on key stages of the project including search terms, research questions, data analysis, and dissemination. A total of 14,142 articles were identified; 66 met the inclusion criteria. We used reciprocal, refutational, and line of argument analytical approaches to identify convergence and divergence between studies. The synthesis showed that current models of statutory mental healthcare are experienced as a major barrier to the delivery of person-centred care to those in ethnic minority groups due to the perceived dominance of monocultural and reductionist frameworks of assessment and treatment (described as “medical” and “Eurocentric”) and direct experiences of racist practice. The lack of socially oriented and holistic frameworks of knowledge and understanding in medical training and services is experienced as epistemic injustice, particularly among those who attribute their mental illness to experiences of migration, systemic racism, and complex trauma. Fear of harm, concerns about treatment suitability, and negative experiences with health providers such as racist care and medical neglect/injury contribute to avoidance of, and disengagement from, mainstream healthcare. The lack of progress in tackling ethnic inequalities is attributed to failures in coproduction and insufficient adoption of existing recommendations within services. Study limitations include insufficient recording of participant characteristics relating to generational status and social class in primary studies, which prevented exploration of these intersections.

**Conclusions:**

In this study, we found that the delivery of safe and equitable person-centred care requires a model of mental health that is responsive to the lived experiences of people in ethnic minority groups. For the people considered in this review, this requires better alignment of mental health services with social and anti-racist models of care. Our findings suggest that intersections related to experiences of racism, migration, religion, and complex trauma might be more relevant than crude ethnic group classifications. Strategies to tackle ethnic inequalities in mental healthcare require an evaluation of individual, systemic, and structural obstacles to authentic and meaningful coproduction and implementation of existing community recommendations in services.

## Abstract 2

In a meta-ethnography, Dr Narinder Bansal and colleagues report insights into ethnic inequalities in mental healthcare in the UK.

## Author summary

**Why was this study done?:**

- People from ethnic minority groups in the UK have poorer mental health and access to mental healthcare, and more negative experiences and outcomes compared to the majority white British group.
- These inequalities have been reported for over 50 years.
- There remains some debate regarding their causes and the interventions required to tackle these.
- In order to develop effective interventions, we need to consider a wide range of perspectives from communities, service users, carers, and mental health professionals in a way that allows interrogation of commonalities and variations in experience across a diverse range of ethnic and lay/professional backgrounds.

**What did the researchers do and find?:**

- We conducted a comprehensive literature search and identified 66 relevant studies that explored the experiences and perspectives of people in different ethnic minority groups and mental health professionals in relation to access to and experience of mental healthcare.
- We identified key themes across papers and explored similarities and differences in experiences across ethnic groups and between communities and mental health professionals.
- We found that current statutory approaches to the assessment and treatment of mental illness are experienced as a major barrier to the delivery of appropriate and person-centred care to those in ethnic minority groups.
- These are perceived to exclude the everyday realities of people’s lives including racism, migration stress, complex trauma, and spirituality and the ways in which these may contribute to (or mitigate) mental illness.
- The lack of progress in tackling ethnic inequalities is attributed to superficial attempts at coproduction and inadequate adoption of existing community recommendations within services.

**What do these findings mean?:**

- The delivery of safe and equitable person-centred care requires a model of mental healthcare that recognises, and is responsive to, the lived experiences of people in ethnic minority groups.
- This includes paying attention to how experiences of racism and migration affect mental health during assessment and treatment and tackling institutional racism in mental healthcare.
- Strategies to tackle ethnic inequalities in mental healthcare require an evaluation of existing barriers and obstacles to authentic and meaningful inclusion of people with lived experience from ethnic minority groups in the development and delivery of mental healthcare services.

## Introduction

Ethnic inequalities in mental healthcare have been documented across the Global North [1–7]. Most of these data come from the United Kingdom (UK) [7,8], where ethnic inequalities in access to, experience, and outcomes of mental healthcare have been reported for more than 50 years [3,7,9,10]. People from ethnic minority groups are more likely to have undiagnosed and untreated mental illness [11–14], come into healthcare via crisis or other aversive pathways [7,15], and receive a diagnosis of severe mental illness [16] compared to the majority ethnic group. These disparate patterns of service access and utilisation incur significant personal and healthcare costs. Delayed provision of care for mental illness is associated with worse outcomes for common and severe mental disorders and increased use of crisis pathways [17,18].

The UK experience is particularly compelling given that healthcare is considered free (at the point of access). There has been a notable absence of progress in addressing these inequalities [19–21], despite numerous policy and legal interventions to improve mental healthcare for ethnic minority communities [22–25]. Explanations offered for the lack of progress include a poor understanding of the nature of ethnic variations in mental health and conflicting professional narratives on the key drivers of these [5,26–31]. This dispute centres around the role of societal factors, such as racism, despite the widespread empirical support for racism and its consequences as a key driver of ethnic inequalities in mental health in the academic literature [32].

A nuanced understanding of how ethnic inequalities in mental healthcare are created and sustained requires interpretative research methods that bring together and interrogate the complexity of experiences of people from diverse ethnic and stakeholder backgrounds including mental health professionals from statutory (services provided by the state) and nonstatutory service settings. This approach can help develop a better understanding of how experiences of racism shape and perpetuate ethnic inequalities in access, experiences, and outcomes [5].

Qualitative Evidence Synthesis (QES) is widely recognised as a valid and rigorous way to consolidate knowledge and develop new conceptual insights from existing literature [33,34]. Meta-ethnography is the most influential and widely used form of QES in healthcare [35]. Unlike other forms of QES, meta-ethnography takes an interpretative, not aggregative, approach to synthesis. This enables conceptual development and theoretical advancement, which is particularly valuable for informing decision-making in policy and practice [33,36,37] including the development and enhancement of clinical guidelines [38]. The aim of our study was to use meta-ethnography to provide a new conceptual understanding of the nature and drivers of ethnic inequalities in mental healthcare. Specifically, we sought to understand why people from ethnic minority groups are underrepresented in primary care mental health service provision and overrepresented in crisis pathways and detention.

## Methods

### Meta-ethnography approach

The meta-ethnography was led by NB (expertise in ethnicity and health/mental health) with supervision from AM (expertise in meta-ethnography and mental health), and support during double/triple screening, data extraction, and interpretation from RC (experience of qualitative evidence reviews and mental health research). We conducted and report the synthesis in accordance with the techniques of meta-ethnography originally developed by Noblit and Hare [39], the recent bespoke eMERGe reporting guidance for meta-ethnographies [35], and Standards for Reporting Literature searches (STARLITE; [40]) developed for qualitative evidence. We followed the seven stages of meta-ethnography methodology as outlined in Table 1 below [35,39].

**Table 1:** Seven stages of meta-ethnography [39].

Stage	Name of stage	Summary of phase
1	Selecting meta-ethnography and getting started	Establishing aim and rationale for meta-ethnography
2	Deciding what is relevant	Search strategy, literature search, and screening
3	Reading included studies	Repeated reading, data extraction, and noting down interpretative metaphors (key concepts)
4	Determining how studies are related	Comparison of study characteristics
5	Translating studies into one another	Translation of interpretive metaphors across studies
6	Synthesising translations	Development of overarching concepts (synthesised translations) and new conceptual interpretation
7	Expressing the synthesis	Summary of findings, recommendations, and conclusions

The building blocks for a meta-ethnography are first-order constructs (verbatim data reported in published papers) and second-order constructs (the themes generated by authors of the published papers based on these data). We used these to develop new interpretations (third-order constructs) by identifying key interpretative metaphors [39]—a key word or short phrase that ignites the reviewer’s imagination and seems to have analytic leverage—relating to our primary research question: Is it possible to explain, using existing research, why people from ethnic minority groups are underrepresented in primary care mental health service provision and overrepresented in crisis pathways and detention? Our definition of first-, second-, and third-order constructs followed the approach taken by previous published meta-ethnographies [41] recognised as seminal by eMERGe guidance [35].

During data translation and synthesis, we used three established meta-ethnography methods of analysis—reciprocal, refutational, and line of argument. These allowed us to explore commonalities (reciprocal) and inconsistencies and exceptions (refutational) across papers and develop a new conceptual interpretation (line of argument) from the data [35]. Given the epistemological tensions and imbalances in this field, particularly between academic spaces and the third sector, we took a multisectoral and “lived experience” approach to our review. This included the inclusion of grey literature and taking a lived experience participatory approach to synthesis, as described below. This helped broaden our epistemological lens to include voices from ethnically and socially diverse service users and mental health service practitioners and gain lived experience feedback at each phase of the work.

### Patient, public, and practitioner lived experience advisory group

We recruited 10 individuals with a range of ethnic backgrounds (self-defining (without any prespecified category options) as white British, white Irish, Bangladeshi, African Caribbean, British Sri Lankan, African), genders (seven females and three males), and experiences of mental healthcare (service user, carer, or mental health professional) to our patient, public, and practitioner advisory (PPPA) group. Professionals included those working in statutory services with experience of delivering services to people from ethnic minority communities (mental health social workers and psychological practitioners), as well as representatives from third sector organisations established to provide culturally tailored mental health services. The inclusion of practitioners is important given their role as key witnesses of clinical practice and care delivery [42]. Some of the practitioners were also service users and offered a dual lens as consumers and providers of services. This included helping provide context around institutional and structural challenges to providing culturally appropriate and equitable care. We held three PPPA meetings during the project. These focused on providing safe and inclusive spaces to allow all participants to voice and discuss sensitive themes and share experiences openly and without fear of judgement. This was particularly important given the inherent power dynamics between service users, providers, and researchers. Meetings focused on the development of the study research questions and search terms, the themes emerging from the analysis, and, at the final meeting, discussion of the synthesis findings and dissemination. Synthesis findings were shared with the PPPA group during two stages of data translation and synthesis. These discussions revealed commonality in the experiences of service users, carers, and practitioners. We also found significant resonance between the lived experience of the advisory group and synthesis findings providing important triangulation to our analysis. Notably, statutory service practitioners were most critical of current service provision and more vocal about the need to adopt decolonised approaches to this field of research.

### Deciding what is relevant

Our university librarian provided advice and guidance on the search strategy and literature search. We included all published and unpublished primary research studies that used qualitative methods of data collection and analysis that were identified using our search strategy. Like others [43,44], we adopted broad search criteria considering empirical rigorousness as well as epistemological strength. For example, some articles may be considered weak in their reporting of methods but epistemologically strong in terms of the data captured. Our approach includes studies that consider individual, interactional, and systemic complexities and are reflexive about processes of knowing, e.g., which actors get to be recognised as those with knowledge of value to policymakers and practitioners, and what evidence becomes incorporated into policy and practice as a result. We (NB) carried out a comprehensive search of electronic databases of published (PsycINFO, Medline, CINAHL, and Social Care Online) and grey literature (OpenGrey and Kings Fund). The inclusion of grey literature was critical given the preponderance of relevant but unpublished literature in this field particularly from the third sector [45]. There was no restriction on year of publication, with database searches including literature published from its earlier period until October 2, 2022. All searches were restricted to a UK setting. Meta-ethnography as a method is not aggregative but an interpretative approach [39]. The interpretative paradigm means that social, cultural, and historical context is more highly valued as a factor in analysis. Our decision to restrict the search to UK healthcare systems ensured comparisons were drawn across consistent healthcare, legal, and policy contexts enabling sufficient resonance to make these comparisons meaningful.

When developing our search strategy, we prioritised sensitivity over specificity. As shown in Appendix 1 (S1 Appendix), we identified relevant subject headings and broad search terms for “ethnic group” (population), “mental ill health” (phenomena of interest) and “help-seeking”/“experience” (study focus) for each database. These broad categories of search terms were combined, and, where possible, we applied a qualitative and UK filter for each database (e.g., Ethnic group + mental ill health + (help-seeking OR experience) + qualitative filter + UK filter). Search terms for grey literature were less specific due to the lack of filters in these online databases. Consequently, we carried out several searches using different combinations of search terms (OpenGrey: “ethnicity and mental health”; “distress ethnic”; “perceptions ethnic”; Kings Fund: “Ethnic and mental health”). Searches were limited to English language studies conducted in the UK. Database searches were supplemented by examining reference lists of key papers and reports (citation snowballing) as well as consultation with experts in the field. Full-text articles were double screened against the inclusion and exclusion criteria by the analytic team (NB, AM, RC).

During full-text screening, we noticed substantial differences in methodology, reporting, and analysis between articles. This included the underreporting of both methods and theory in some academic papers. It was unclear the extent to which this was due to space constraints for qualitative research papers in academic journals and how much was due to omissions in the research and reporting process itself. Like others [41,46], we took the position that, for meta-ethnography, conceptual and theoretical utility may be more important than the quality of reporting. Our initial screen considered conceptual richness, theoretical utility, and epistemological strength. We adapted the CASP (Critical Appraisal Skills Programme) [47] critical appraisal tool to include assessment of epistemological strength and conceptual richness. Conceptual richness is a key inclusion criterion for meta-ethnography and as a term refers both to evocative images and ideas in verbatim data as well as author themes that move beyond thin description. A conceptually rich data set is one that shows potential to contribute significantly to an interpretive meta-ethnography because the authors have demonstrated intellectual curiosity and thematic mastery. Assessing whether data are conceptually rich involves an interpretative process that relies upon the reviewers’ professional judgement and expertise as qualitative researchers. The purpose of the critical appraisal was not to exclude articles but to get a sense of variations in quality and relevance and consider the appropriateness of the primary studies for meta-ethnography synthesis.

**Fig 1:**
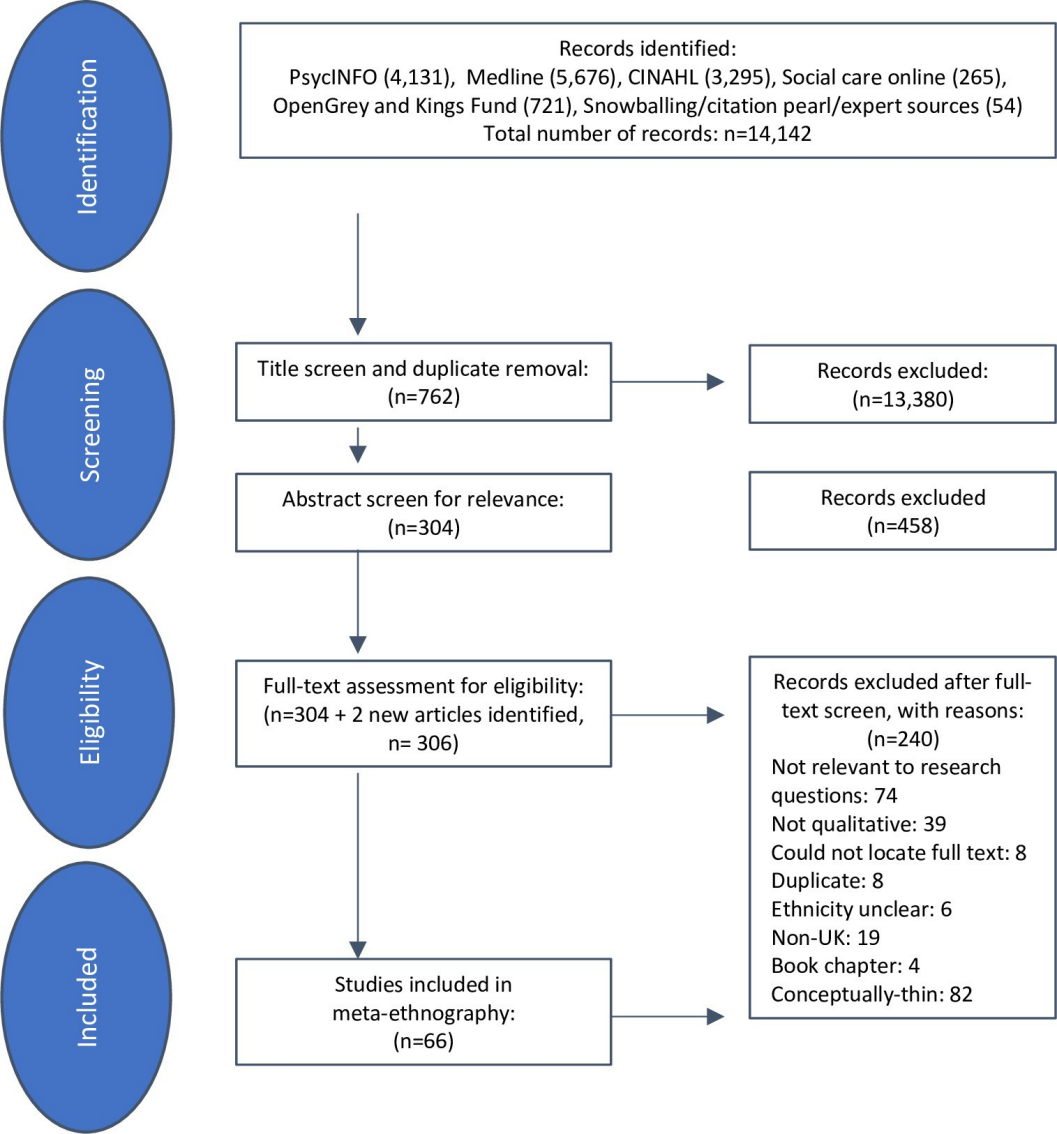
Outcome of study selection.

Our search strategy identified 14,142 articles (Fig 1). These were uploaded to Covidence (https://www.covidence.org/) and title screened for relevance by the lead investigator (NB) resulting in the identification of 762 titles relevant to the topic. These titles were then abstract screened for relevance, with 20% double screened by AM. During title and abstract screening, we took an inclusive approach excluding papers only if it was clear from the title and abstract that these articles were not related to ethnicity and mental health. Where there was any uncertainty, papers were included and moved to full-text screen. We found that our broad search criteria cast a wide net leading to the identification of a large number of articles that were unrelated to mental health and ethnicity. We found a large number of descriptive papers that failed to meet the key inclusion criteria for conceptual richness for meta-ethnography. We included all conceptually rich [39] primary research studies on ethnicity and mental health that used qualitative methods of data collection and analysis. We excluded studies that focused solely on causation of mental illness in ethnic groups. There were no restrictions in terms of setting (i.e., voluntary/statutory, community, primary, or secondary care) or type of mental illness or symptoms. This allowed us to include broad conceptualisations of distress and help-seeking and the large body of research in this field exploring barriers to service access among those who had not utilised formal services (hereafter referred to as community or communities). Identification of two additional articles from the bibliographies of these papers led to a final selection of 306 articles for full-text double screen. We could not locate the full text of eight papers in the time frame of our study despite exhausting routes such as contacting study authors and libraries. As shown in Fig 1, this full-text screening established that 66 articles met the inclusion criteria for our study.

### Analysis

#### Reading and data extraction approach

Noblit and Hare refer to this stage as “reading included studies” (stage 3, Table 1) [39]. The analytic team (NB, AM, RC) familiarised themselves with the content and detail of all the included studies through close and critical reading and rereading of papers. The formal process of identifying constructs and key concepts started during the data extraction process. First- and second-order constructs were extracted initially by NB for all 66 papers. AM and RC then added either new first- and second-order constructs to the same table or interpretative reflective comments following their own review of the papers. In this way, data extraction was an interpretative conversation, with different disciplinary perspectives prompting questions and sometimes conflicting interpretations of the same piece of data. These were resolved during team discussions. Third-order concepts were mostly grounded in first-order concepts (verbatim data) or language cited in the original articles (second-order constructs). The first 24 papers contained interpretations from all three researchers (triple interpretation). We were satisfied that there was sufficient resonance within the team to move to double interpretation for the remaining 42 papers, with AM and RC carrying out 50% of second layer interpretations each. During screening, we noticed that Edge and colleagues had produced a number of conceptually rich papers, where a common focus and sample—on perinatal mental health in African Caribbean (AFC) women—had enabled the evolution of certain themes, focus, and nuance over time [48–51]. We used these insights to begin by extracting data from all selected articles focusing on experiences of women in order to explore whether similarly gendered experiences could be identified for women in other ethnic groups. We continued by extracting according to our prespecified demographic clusters (informed by advisory group discussions and suggestions), with papers focusing on men, young people, then by specific ethnic group. Where possible, we crudely clustered data extraction by ethnic group to get a sense of similarities and differences in experiences across our data. Qualitative research does not seek to generalise experiences and it was not the intention of this study to make generalisations or assumptions about the experiences of specific ethnic groups. However, we took note of sample characteristics and considered whether there were any important commonalities in the experiences of participants from the same ethnic group across studies. Some papers contained mixed samples of people from ethnic minority groups commonly identified as BME (black and minority ethnic) and others combined experiences of African Caribbean, African, and black participants.

#### Translation and synthesis process

We carried out data translation (stages 4 and 5, Table 1) in stages to help preserve the context and meaning of relationships between key concepts within demographic clusters (age, gender, and ethnic grouping). We mapped relationships between emerging metaphors in each paper, noting similarities and differences between and within clusters. We followed an iterative process of mapping each emerging metaphor across all articles retrospectively and prospectively, creating individual data extraction forms for each article and creating a grid in Excel of first- and second-order constructs by study and key metaphor [41]. We used this grid and iterative phases of data mapping to synthesise the data presented in all the studies, using reciprocal, refutational, and line of argument approaches (stage 6, Table 1). During this process, we paid attention to any emerging major trends relating to wider study characteristics such as geographic region, time of publication, and clinical setting.

Accounts that appeared refutational were discussed within the team and advisory group, where we explored the nuanced and broader narrative relating to each account and the extent to which these might indicate important explanations for ethnic inequalities in mental healthcare. Some members of the advisory group thought that some of these metaphors could also apply to white middle class service users (particularly “it’s useless” and “battling against”) and suggested that perhaps the data were highlighting problematic service structures that affected all service users. This led to much discussion and debate about the extent to which metaphors were universal and a wider exploration of the emerging relationship between oppressive experiences and negative service responses. The advisory and analytic group thought that it was important to highlight both issues (problematic services structures potentially affecting all ethnic groups and the role of oppression in creating ethnically differentiated experiences for minority groups) in the findings. During this process, we found insufficient evidence that differences in mental health literacy was an important explanation for variations in access, experiences, and outcomes between minority and majority ethnic groups [52,53].

## Results

### Study characteristics

Summary study characteristics of the included articles are shown in Table 2. Articles were published over a 23-year time period (1999 to 2022), mostly in academic journals (56/66). Papers focused on the experiences of adults and young people and a mix of service users/carers (*n* = 21), communities (*n* = 18), service providers (*n* = 7), with some articles including information from a combination of lay, service user, and provider perspectives with experience of both statutory and voluntary sectors (*n* = 20). Studies focusing on service experiences were mostly located in secondary care (*n* = 27). Articles included information on the experiences of people from 14 different ethnic groupings from 15 UK cities/regions. A list of all included papers is provided in the supplementary table (S2 Appendix).

**Table 2:** Summary study characteristics of included articles.

Study characteristic	N
Age grouping Adult Young person Mix of adult and young person	50 11 5
Publication year 1999 to 2003 2004 to 2008 2009 to 2013 2014 to 2018 2019 to 2022	10 12 15 15 14
Publication type Academic journal paper Reports Postgraduate thesis	56 5 5
Research sector Academia Multisectoral Third sector	53 8 5
First author ethnicity Unknown BME White	28 29 9
Participant background Mix of lay/service user/provider/statutory/voluntary Community/lay Service users/carers Service providers	21 18 21 6
Study setting Community Statutory service Community and service Statutory and nonstatutory services Nonstatutory service	27 21 8 8 2
Statutory service setting Secondary *Adult* *Young person* Primary and secondary adult Primary	20 (*16)* *(4)* 5 4
Ethnic groups studied BME/Heterogenous ethnic sample Black African/African Caribbean/black South Asian Chinese Refugee/Asylum seekers Pakistani Somali Gujarati Jewish Nepalese East African Muslims Polish Central/Eastern Europeans Nepali and Iranian	15 20 8 5 6 3 2 1 1 1 1 1 1 1
Generation Not reported First Mixed sample of multiple generations Second/UK Born	36 18 9 3

BME, black and minority ethnic; UK, United Kingdom.

### Key interpretative metaphors

We identified 12 interpretative metaphors relevant to our research question. Table 3 shows how these metaphors relate to second-order constructs and provides a summary of each construct, using the language and key phrases of the authors and study participants where possible. Metaphors are listed in a temporal sequence categorising key transition points relating to help-seeking, access, experience, outcomes, and desired changes. These are categorised as:

1. Practical barriers that prevent or stall help-seeking or help-giving, which relate to the shortage of resources (Metaphor: “It’s not possible”)
2. Fears and concerns about service access and provision that relate to anticipated costs and benefits (Metaphors: “Not allowed”; “Not worth taking the risk”; “Epistemic tensions”; “It’s useless”; “Turned all the stones”)
3. Experiences and consequences of service use (Metaphors: “Battling against”; “Turning away”; “Turning towards”; “Falling through the net”; “Victim of the system”)
4. Desired solutions and changes to mental health services (Metaphor: “Business as usual is not enough”).

**Table 3:** Translation of second-order constructs into key metaphors (third-order interpretations).

Temporal sequence used to frame line of argument	Key metaphor	Second-order constructs	Summary definition (translation) of the second-order construct	Papers that include the second-order construct
1. Practical barriers to access and provision	Not possible	Linguistic communication barriers	Language identified as major institutional barrier to providing culturally sensitive services. Includes lack of practitioners from same linguistic background and low availability of interpreters particularly in inpatient settings and for smaller and more recent ethnic minority groups.	[52,60,69,74,75,84,87,89,101,102,111]
		Financial constraints and hierarchy of needs	Not possible to find services or explore alternative treatments like private therapy due to economic circumstances of ethnic minority groups from socially disadvantaged communities and need to attend to primary needs—food, housing, work, childcare.	[57,69,83]
		Absence of time	Staff lacking time to explore religious and cultural needs with patients.	[111]
2. Fears and concerns about service access and provision	Not allowed	Not allowed to have mental illness	Intergenerational transmission of need to survive/be strong (not have illness) due to systemic racism, intergenerational trauma, and historical oppression (slavery, oppressive regimes and “racial battle fatigue”). Includes the development of racialised identities and “defensive cultural moves” that do not allow space for vulnerability.	[48,49,65,67,69,71,78,94,127,128]
			Not considered a real illness by community (lack of legitimacy).	[78,104]
			Mental illness associated with personal failing, lack of faith in God or adherence to religion (illness not allowed by God, faith, or self).	[69,78,88,102,103,105,128]
		Not allowed to disclose illness/seek help	Not allowed by family members to disclose illness.	[64,76,78,83,91,94,98–101]
			Not allowed by religion or culture.	[54,63,127]
			Not allowed to burden female practitioners with distress.	[81]
			Not legally allowed to access services.	[74]
	Not worth taking the risk	Fear of cultural and social pathologisation	Fear that personal beliefs and social problems would be pathologised as illness in services.	[83,85,87,89]
		Fear of spoiled identity and social isolation, exclusion, or social death	Fear of being assigned a stigmatising identity. Avoidance of services linked to preservation of identity and fear of wider negative social and economic consequences of spoiled identity. In some accounts, perceived negative consequences extend to families and communities.	[49,69,75,78,81,88,89,91,93,95,101–103,106,107]
		It’s not safe to leave the house.	Experiences of racism and crime act as a barrier to leaving the house with preference of seeing professional in own home.	[54]
		Fear of confidentiality breaches	Fear of confidentiality breaches in clinical encounters particularly if practitioner is from the same community.	[78,98,100]
		Fear of systemic racism	Anticipation of racist stereotyping and treatment within services discouraged formal help-seeking. Services associated with criminalisation of black people. Also concerns about Islamophobia. Experiences of racism and oppressive racial stereotypes attached to ethnic groups serve as powerful disincentives to approaching services particularly for people facing multiple layers of discrimination based on race, gender, class, and migrant status. Refugees and migrants perceive that they are stereotyped as “liars” and “scroungers.” Black British women also feel that they are seen as scroungers and avoid services in an effort to resist fulfilling racial stereotypes “an alien stealing our money.” Black men experience multiple stereotypes “weed smokers, violent, aggressive, schizophrenic.”	[57,63,65,67,71,74,93,94]
		Fear of medical harm	Not feeling safe within service. Mistrust of doctor and fear of consequences of treatment, mainly medication “zombies,” coercive treatment, and retraumatisation with talking therapy for refugees. Services seen as a form of social control. Fear that help-seeking would lead to the death of a loved one due to police intervention or confinement in hospital. Concern that “you go there when you are the most vulnerable and they make mess of it.”	[48,56,58,60,66,69,74,80,101,106,109,110,127,128]
		Linguistic and conceptual communication barriers	Not daring to access service due to awareness of linguistic and conceptual communication barriers	[86,101]
		Lack of cultural diversity	Perceived unavailability of practitioners from own ethnic background a barrier to accessing services.	[48,74]
		It’s not safe to talk about race	Professionals do not feel safe talking publicly about issues of race and culture within staff teams. Maintaining “air of secrecy” with conversations hidden “behind closed doors.”	[56,58]
		It’s not safe to talk about religion	Staff fear losing professional identity and facing exclusion from the British medical community for talking about religion and spirituality in a clinical setting. Asking patient about religion perceived to be a taboo subject within staff teams.	[92,111]
		Fear of social service involvement	Participants discussing perinatal mental health discussed family fears that disclosure of illness would result in removal of baby by social services.	[128]
	Epistemic tensions	Social causes of illness	Participants make sense of distress as originating from social risk factors including racism, discrimination, isolation, trauma, and migration stress. Depression is a new word, a western disease, associated with migration and life in the West (i.e., social isolation, loss of status).	[63–67,74,78,79,85,90]
		“Alien” services: Western “medical” models of illness conflict with social and holistic explanations	Clash between individual illness explanations and medical diagnosis. Not a mental illness but a response to social conditions and systemic trauma. Refusal to accept biomedical diagnosis or treatment, perceived as an inappropriate medicalisation of social or spiritual causes. Holistic constructions of mental health do not align with “old fashioned” Western model of psychology where there is an “artificial” separation of body and mind and where illness and treatment is located in the individual. Mental health service approach described as “alien” and “profoundly inadequate.” Includes use of words such as psychosis and depression.	[49,54,56,67,69,75,78,80,84–90,95,101]
		Epistemic rights and authority	Recognition of doctor as authority figure with primary epistemic rights	[81]
			Denial of authority of medical staff to diagnose a condition as mental illness	[106]
		Colonisation, orientalism, and “Eurocentric”/racist knowledge	Related to colonisation of knowledge including “orientalism” and racist discourse within staff teams, services, and research. Cultural knowledge within services and research perceived to be constructed on racial and cultural stereotypes where Western culture is superior to “repressed” inferior Eastern cultures. Includes the systemic focus on “exotic quirks” in research, rather than racism and trauma, as creating differential experiences across ethnic groups. And the presence of racial stereotypes and cultural assumptions in services and exclusion of non-western knowledge and belief systems. Perception that services are epistemologically “white”/ “Eurocentric” and not able to attend to the needs of people from ethnic minority groups.	[55–58,71,92–94]
		Spiritual explanations versus biomedical	Tensions between spiritual and biomedical epistemologies. Spiritual healers and faith leaders treated with respect, perceived to have epistemic authority. Spiritual explanations perceived to be associated with low mental health literacy and older generations.	[66,78,85,87,89,94]
		Crossing the epistemic threshold	Knowing when to cross the threshold from seeking help from priest to seeking medical help.	[88]
	It’s useless	Unhelpful and inadequate “medical” approaches	Help-seeking perceived and experienced to be “useless” given reliance on Western biomedical model and approach (“Mechanical,” “Waste of time,” “old-fashioned”). Dominant reasons for this include lack of holistic approach and lack of attention to “root” social causes of illness during assessment and management and “tunnel-visioned” focus on symptoms and medication and not enough attention to patient context, narratives, and experiences.	[64,66,67,69,74,79,87,89,91]
		Overreliance on medication prescription	Perceived and experienced to be the predominant or only treatment option. Medication perceived or experienced to be an ineffective and inappropriate treatment for psychological distress/illness. Lack of experienced benefit “still the same person with same problems.” Considered to be “irrelevant at best and damaging at worst.”	[48,51,67,74,79,84,128]
		Lack of cultural understanding in services	Avoidance due to perception that western doctors will not understand alternative conceptualisations of illness	[64,84,85]
		Useless to seek help given return to traumatic circumstances	Talking about problems doesn’t help when you have to go back home to toxic life conditions.	[77,80]
		Clinical encounters are inadequate	Prior negative experiences (i.e., feeling misunderstood, experiencing low empathy) of clinical encounters influence future help-seeking. Narratives describe difficulties in building a rapport and trusting relationship with GP and communicating distress in 10-minute consultations: “these aren’t 10-minute conversations you can have with someone!” GPs considered to be unsuitable for conversations about emotional distress—too stressed, will just give you tablets, not considered to be “qualified enough” to deal with emotional distress.	[54,69,78,82,89,110,128]
		Lack of power to enact change	Perception that power inequities between the third sector and mainstream services will not allow meaningful coproduction of service change.	[59]
	Turned all the stones	Last resort	Coming into services as a last resort, once all the “stones have been turned,” the “last straw,” “last place to go,” when all other attempts have failed, at the “end of my rope” and there is “nowhere else to turn.”	[51,56,69,76,89,107]
3. Experiences and consequences of service use	Battling against	“Eurocentric” and “medical” services	Frustration at inability of services to deal with language, cultural, social, and ethnic differences and provide culturally capable person-centred care “one size fits all.” Includes lack of cultural sensitivity and inclusion of alternative conceptualisations of mental distress that fall outside the “Euro” and “medical” model. Perception that issues of race and culture do not fit into the biomedical model. Lack of understanding of culturally relevant frameworks of illness and recovery. Biomedical labels and words experienced as stigmatising. Psychiatry appeared synonymous with “white doctors.”	[56,63,64,67,69,71,79,81,84,87,89,99,103,109]
		Oppressive services	Continued dominance of Eurocentric services and biomedical model of care and lack of acknowledgement, acceptance, and incorporation of social and cultural models of illness into services. Location of pathology in individual and systemic denial of role of structural factors including racism and systemic oppression. Lack of holistic care, provision of talking therapy, and exclusion of patient, family, and carers from care planning perceived and experienced as oppressive and major barriers to recovery and healing. Experiences of testimonial injustice include patients, families and carers not being taken seriously by professionals. Concerns dismissed sometimes with tragic consequences. Not feeling “believed” when talking to professionals. Perceptions among staff that narratives from BME groups cannot be trusted or relied on. Experience of inpatient care described as disempowering and “a complete psychological, mental rape.”	[48,55–57,59,61,64,67,69,79,87,92,109,128]
		Overreliance on medication and coercive treatment “symbolic violence”	Participants frustrated, perplexed, and distressed by inflexible medication-focused services. Including anger at the lack of control over medication. Use of force and depot medication described as systemic “symbolic violence.” Lack of information on medication purpose, indication, and side effects. Coercive treatment experienced as dehumanisation. Perception that coercive treatment is imposed as a form of social control of deviant cultural behaviours. Frustration and despair at the trial-and-error approach to diagnosis and treatment. Perception that doctors do not know what they are treating due to perceived lack of thorough clinical assessment, lack of experienced benefit of medication and changing diagnoses. Medication regimes experienced as ineffective and damaging.	[67,79,87,112]
		Retraumatisation	Feeling overwhelmed and retraumatised in service—feeling forced to talk about traumatic experiences when not feeling ready or safe. Inadequacy of current services in dealing with complex trauma.	[56,60,64,69,74,75,77,80,81]
		Inequitable treatment	Perception and experience that BME patients less likely to be offered talking therapy and more likely to be offered medication than white British. Talking therapy desired/requested but not offered. Perception of increased staff violence towards black patients. Service provider perception that services do not believe that black people can be treated with talking therapy.	[57,61–63,79,85]
		Cultural pathologisation and racial stereotyping	Presence of racist discourse, negative cultural assumptions, cultural pathologisation and racial stereotypes within services. Frustration at inadequate clinical assessments and hasty diagnoses that were seen to pathologise cultural behaviours, beliefs, and race. Staff feeling powerless to intervene and correct cultural misunderstandings and racial stereotypes. Religious beliefs pathologized as “an unhealthy obsession and potentially delusional.” Perception and experience that the white middle class presentation is the acceptable patient presentation. Experience that white doctors do not understand where ethnic minority patients are coming from and have a tendency to overmedicate and misdiagnose.	[55,56,58,61–63,67,87,95,111]
		Adversarial interactions	Clinical encounters experienced as adversarial by staff and patients/carers. Staff perceive lack of trust of service by families and carers and families experienced as hostile. Families/carers feel like they have to “battle” to be seen and “shout” to be heard, “know what your rights are” “you really have to fight” because “nobody listens to you when you talk civil to them “and “it is always a struggle to be taken seriously.” Perceived to be a self-fulfilling prophecy for black people “a losing battle.” Families and carers thought that they were perceived as aggressive by staff.	[56,61,74]
		Poor listening	Getting distressed/frustrated because doctor is not listening/not feeling listened to.	[51,61,67]
		Linguistic challenges	Unable to express self to doctor adequately in English and unable to understand doctor especially medical jargon.	[84]
		Business as usual	Frustration that nothing has changed on the ground despite numerous reports and pieces of research. Perception that there is a continued focus on research and no attention to race and equality issues in service senior management. Lack of authentic engagement by services with community stakeholders and/or adoption of community suggestions to allow meaningful change. Perception and experience that statutory services hold all the power and are unwilling to genuinely engage with community participatory initiatives beyond tokenistic. Attempts at improving cultural sensitivity seen to be superficial/inadequate related to the continued dominance of the “Eurocentric” model, “privileged middle class white male” seniors and decision makers, and unwillingness of the “system” to change: “European services dressed up in rice and peas.”	[59,79,87,95]
		Institutional racism	Service provider experience that it is more challenging for ethnic minority service providers to fit into and work in mental health services perceived to be institutionally racist.	[95]
	Turning away	Labelling or medicalisation of social problems	Turning away from service to avoid “labels” and pathologisation of “real” trauma/social problems and lack of attention to root social causes.	[48,64,83]
		Negative clinical encounters	Avoiding services due to previous negative clinical encounters including experiences of poor listening, autocratic communication, insensitive or uncaring staff, testimonial injustice, abuse, misinterpretation, and disempowerment resulting in feelings of frustration, mistrust, and fear. Staff thought to be insensitive or uncaring.	[51,56–58,67,85–87,99,100,103,112]
		Culturally insensitive approaches	Experiences of low cultural understanding and attunement in services.	[89,99]
		Ethnically dissimilar practitioners/therapists	Ethnically dissimilar practitioners/therapists experienced negatively—ineffective and sometimes increased distress due to low cultural understanding/capability and culturally inappropriate advice.	[71,99]
		Ethnically similar doctors/practitioners	Professionals living and working within the same ethnic community (i.e., South Asian) who share a similar cultural background and values as their patients are taken as being “insiders,” whose potential for offering help or support is limited.	[71,98,100,101]
		Retraumatising approaches	Experiencing being forced/pressured to talk about trauma as retraumatising. Turning away internally from therapists and clinicians.	[74,82]
		Lack of continuity of care	Seeing different doctors at each appointment and having to repeat the same thing.	[58,74]
		Lack of improvement/recovery	Lack of improvement in symptoms and uncertainty about efficacy. Treatment thought to be counterproductive. Absence of recovery.	[56,77,84]
		Informal support	Not wanting to burden family members. Avoiding negative reactions from families including their distress and being viewed as weak. Turning away from members of own community due to systemic trauma and erosion of social and moral order due to experiences of war.	[69,75,76,89,103]
	Turning towards	Ethnically similar doctors/practitioners	Seeking out ethnically similar practitioners. Perception and experience that ethnically similar practitioners are more culturally sensitive and better able to understand cultural context. Help from practitioner from own culture experienced to be key to recovery due to shared cultural and spiritual perspective.	[54,64,69,78,85–87,93–95,99,101,109]
		Ethnically dissimilar therapists	Seeing a non-Chinese therapist experienced as beneficial and essential to talking openly without being subject to cultural taboos and constraints.	[99]
		Linguistically similar doctors/practitioners	Seeking out doctors who can speak the same language.	[84,99]
		Nonjudgemental, understanding, and culturally capable practitioners	Ethnicity of practitioner not important if practitioner is open-minded, nonjudgemental, trustworthy, and is able to understand cultural context and nuances, and is culturally capable, respectful, and sensitive. “It depends on the person.”	[63,67,68,78,99,100,128]
		Therapist of same gender	Talking to someone of opposite sex about distress and personal problems considered culturally and socially inappropriate.	[54]
		Creative expression and storytelling	Full recovery “heal my heart” associated with joining singing group and releasing feelings. The act of sharing stories and illness narratives experienced as a key part of recovery. Engaging with and making sense of the illness narrative through creative expression helps transform illness experience (normalising/humanising) Stories allow and open the door to healing. Participation in qualitative research experienced as therapeutic—in some cases, the first-time participants felt listened to. Creativity experienced as a symbolic tool for expression and healing.	[64,65,75,84,87,112]
		Nature	Service users experienced nature as therapeutic and nonstigmatising “nature doesn’t judge you,” “healing is to help ground people and to go through natural things.”	[70,87]
		Medical pluralism	Trying everything. Seeking traditional medicine when Western medicine is not helping.	[86,107]
		Faith and spirituality	Turning towards traditional/spiritual healers for diagnosis and/or treatment. Spiritual healers and faith leaders treated with respect, perceived to have epistemic authority, and talking to them experienced like counselling, perceived to be better able to relate to people than monocultural services. Religion viewed as being fundamental to understanding and managing distress “Praying can help me to calm down.”	[66,78,85,87,89,101]
		Third sector support organisations	Holistic community services that centre around the social model of illness and focus on empowerment and “well-being” and don’t use the word “mental” are highly valued and utilised. Location of suffering and illness experiences in sociocultural context experienced as an important antecedent to healing and recovery. Participating in community support groups and meeting others with similar experiences and social struggles found to be therapeutic. Includes third sector BME community groups that acknowledge and are responsive to experiences of racial and social oppression.	[54,64,65,67,74,83,84,87,98,102,110]
		Empowering political movements and activism	Turning to anti-racist and survivor movement groups for solidarity, experienced as empowering and an important part of the recovery process.	[64]
		Informal support network	Close friends and family first port of call when participants could not resolve difficulties alone. Some younger participants preferred friends over family, appears to be dependent on relationship with parents. Shared values and interdependence viewed positively. Informal social network provided sense of safety and helped maintain privacy (avoiding gossip from wider community).	[63,69,78,89,102,108]
		Talking therapy and trauma-informed therapy	Desiring to talk to someone and be heard. Experiences therapy positively: positive relating, listening, validation. Trauma-informed approaches experienced positively by refugees “she saved my life.”	[54,67–69,75,82,85,90,108]
		General Practice	GP setting considered to be less stigmatising than psychiatric hospital. Relationship with GP felt safe and trusting.	[60,69]
		Invisible places	Schools and colleges recognised as safe invisible places for accessing therapy and support by young people and practitioners, located away from the gaze of the community, reduces risk of spoiled identity (see “not allowed,” and “not worth taking the risk”). Online support allowed young people to connect with others anonymously.	[98,100,108]
		Medication	Medication was seen to be preferable to talking therapy, which was experienced as retraumatising, medication helped them forget their trauma and lifted their mood.	[81]
		Peer support group	Attending a peer support group made women feel like they were not alone and provided a powerful and supportive community.	[128]
		Rest, diet, and exercise	In terms of recovery, participants describe nurturing self/learning to relax and self-care as helpful to preventing a bad day spiralling into a crisis. Finding a rhythm by taking walks, cycling, swimming, immersing in photography.	[90]
	Falling through the net	Clinician not recognising mental ill health	Service providers failing to recognise emotional distress/mental illness leading to service users not receiving appropriate and timely support.	[48–50,56,57,71,74,84,85,100,105,112,128]
	Victim of the system	Feeling silenced and dismissed	Experiences of patients, families, and carers not being taken seriously by professionals. Concerns dismissed sometimes with tragic consequences. Not feeling “believed” when talking to professionals. Perceptions among staff that narratives from BME groups cannot be trusted or relied on.	[51,55,56,61,67,79]
		Symbolic violence	Services experienced as disempowering, paternalistic, condescending, and described as autocratic, dehumanising, and a form of social control. Inflexible, coercive, ineffective medication-focused treatment and service with severe side effects and no improvement in illness. Extensive use of medication with no alternatives offered. Medication experienced as a form of symbolic violence.	[56,61–64,67,79,87,112]
		Misdiagnosis	Accounts highlighting misinterpretation of cultural beliefs and experiences as illness by service providers.	[56,67,87,95,111]
		Verbal and physical violence and injury	Accounts of bullying, verbal aggression, physical beatings, and injury within services. Includes threats of coercive treatment, seclusion and physical violence “injections” and “grabbing” in wards. Physical damage and pain caused by restraint measures.	[56,61–63,74]
		Deterioration in mental or physical health	Service experiences exacerbate distress and negatively impact mental or physical health. Includes a downward spiral of decreasing mental and physical function with increasing use of coercive treatment and medication side effects “zombies” and no obvious improvement or treatment response. Coercive treatment experienced as making mental illness “worse.”	[56,61,67,75,77,79,80,112]
		Systemic oppression and injustice	Service response to black people perceived to mirror and reenact societal oppression experienced in other institutions (education, employment, criminal justice system). “You come to services disempowered already.” Experience of increased staff verbal and physical violence towards black patients and violent induction of black people into mental health services.	[56,61,74]
4. Desired solutions and changes to mental health services	Business as usual is not enough	Need better access to holistic healing and creative therapies	Desire for access to safe spaces for exploring and making sense of distress. Includes better access to healing therapies, natural spaces, peaceful environments, creative therapy and activities and a holistic recovery program. Need for better access to trauma-informed therapy and talking therapy.	[56,57,64,65,67,74,75,85,87,88,100,112,129]
		Heal and end systemic injustice and racism against black community	Need for services that are sensitive to impact of racial oppression on mental health. Need for independent advocacy and empowerment of oppressed ethnic groups and intersections, build capacity, strength, and resilience within communities. Develop authentic, empowering, and effective partnerships with community organisations across sectors and show genuine willingness and potential for services to change. Remove obstacles to change in community and services. Requires explicit removal of institutional racism from services with clear demonstration to community of removal. Get to the root of the problem: tackle economic disadvantage and racial discrimination in employment sector. Need for black role models in schools and positive media presentation.	[56,57,59,64,65,74,88,93]
		Need a paradigm shift	Request for humane, dignified, respectful services that take a democratic and person-centred approach. Move away from a traditional “Eurocentric” and “medical” model and drop the labels “mental”/“depression.” Need for a service model that is responsive to social causes of illness including social exclusion, oppression, and discrimination and pays attention to “expert” patient narratives of illness. Desire for more democratic clinical encounters and social interventions. Integration of “Western” biomedical model of treatment with spiritual/cultural/faith knowledge systems. Location of support in community/existing community assets and development of community “gateway agencies.” Recognise third sector expertise and provide political and financial support to, collaborate with, community BME and other voluntary support organisations. Identification of the need for epistemic brokering, collaborative care planning, and negotiation “meet in the middle.” Build infrastructure to allow coproduction of services that are meaningful to BME communities including shaping professional training by service users and BME communities. Consider limitations of “Eurocentric” research methodologies and traditional “Western” therapeutic models and assessment approaches in understanding complex multicultural worldviews.	[52,54,56,57,64,66–69,71,74,75,79,81,83,85,87–89,92,110–113]
		Training and resources	Increase ethnic diversity within services. Need for genuine investment in the development and empowerment of BME staff. Beware of naïve conceptualisations of culturally competent services. Mainstream health and social care should be supported to build their capacity to make accurate person-centred assessments of the mental health needs of people from ethnic minority communites and commission a diverse range of suitable services. Leadership, particularly leadership skills of black staff (“bring in the Africans”), should be developed. Create safe spaces for conversations and discussions about race within services—staff teams and clinical encounters. Current equality and diversity training is not enough. Staff lack confidence in dealing with cultural diversity and asking patients about religious and cultural needs. Usual approach to care does not meet unique needs of refugees and asylum seekers.	[55,56,60,77,81,82,88,92,98,109,111]

BME, black and minority ethnic.

The organisation of metaphors in these categories does not imply linearity. However, this approach provided a valuable starting point for organising the data, particularly in terms of understanding the relationships between access, experience, outcomes, and desired changes.

### Reciprocal and refutational synthesis

The reciprocal synthesis found strong resonance in the experiences of study participants in relation to the key metaphors. These metaphors were meaningful regardless of gender and age groups, across long established ethnic minority groups in the UK, more recent migrants, and refugee groups, and across communities, service users, and providers. The perceived dominance of a biomedical and “Eurocentric” model of mental healthcare and exclusion of alternative epistemological frameworks was identified as a powerful theme that cut across metaphors relating to access, experience, outcomes, desired solutions, and barriers to progress. The current system of care was perceived to be designed for people from white British backgrounds and experienced as meaningless, oppressive, and outdated. This related to the perceived reduction of individuals to labels and symptoms and exclusion of social, racial, religious, and other cultural aspects of illness and person during clinical assessment and treatment. This is discussed in detail in the line of argument synthesis below. An important common theme across ethnic groups in terms of positive experiences was that participants were seeking a safe space to be seen, heard, and understood in the context of their lived experience without judgement or bias. In this context, nonstigmatising and expressive modes of therapy were highly valued including talking therapy and creative therapy.

A nuanced analysis of accounts that appeared refutational revealed universal themes in relation to the importance of good supportive relationships, trust, and accommodation of cultural diversity in the provision of support for mental illness. It was difficult to make generalisations about the experiences of a specific ethnic group given the nature of sampling in qualitative research and lack of information on participant intersections (i.e., by generation, social class, social deprivation). Moreover, this was not the intention of our analysis as this is generally an inappropriate use of qualitative methods. However, we did note that certain themes were more powerful for some groups than others. For example, the impact of systemic racism and oppression was a particularly powerful theme in research focused on African Caribbean/African/black groups. Services appeared to be most challenged in meeting the needs of ethnic groups who had complex needs (including severe trauma) and fragile social circumstances (unemployment, housing difficulties) such as refugees and asylum seekers. We found that the most distressing accounts of service use were located in narratives from these ethnic groups. While clinical diagnoses were experienced as oppressive across all ethnic minority groups, the storylines differed depending on the extent of social oppression and marginalisation experienced by individuals and communities. In this context, we identified important intersections relating to experiences of racism, social marginalisation, migration status, the role of faith and spirituality in a person’s life, English language literacy, experiences of complex trauma, fragility of social circumstances (including employment, housing), and the presence of supportive informal networks.

### Line of argument synthesis

**Fig 2:**
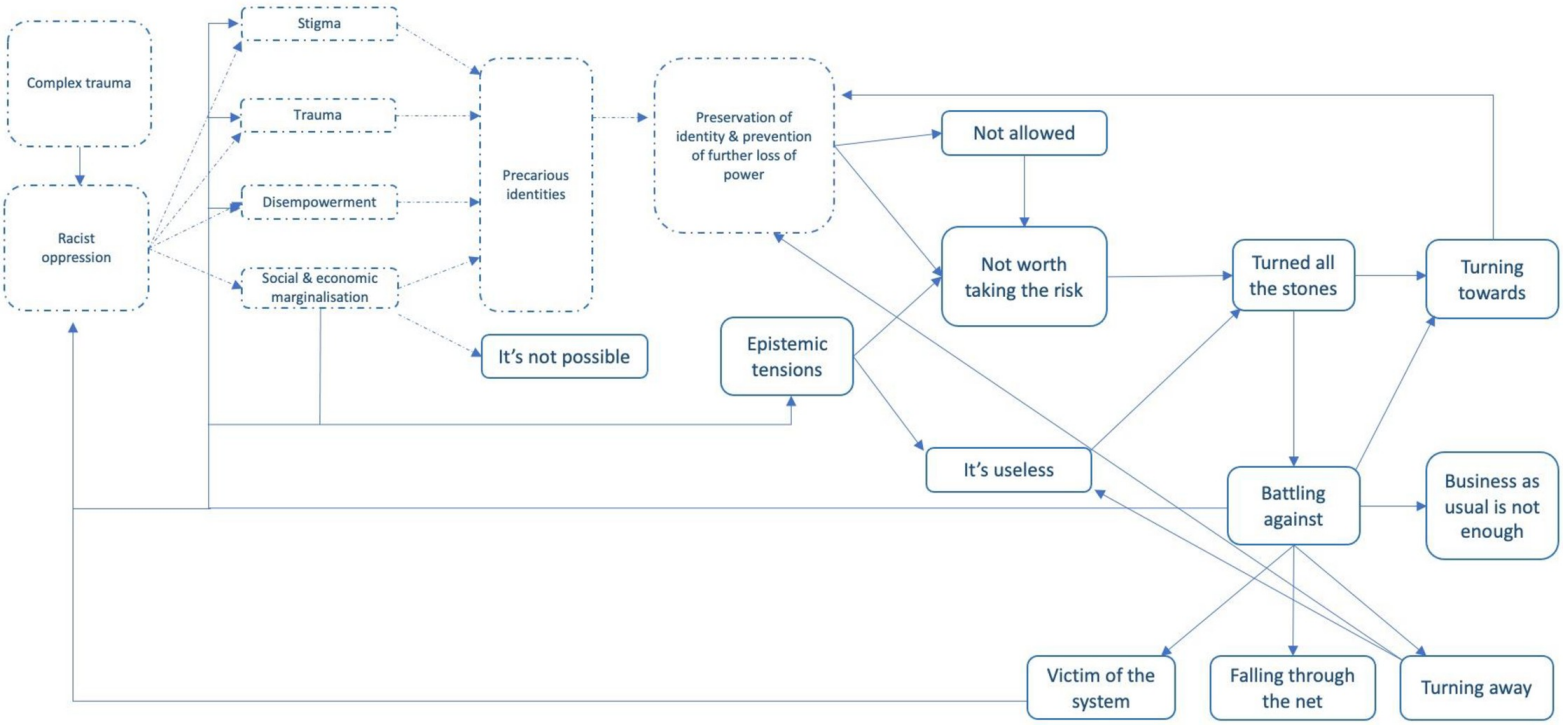
Line of argument synthesis. Dotted lines used to distinguish social determinants and processes from key metaphors (boxes with solid lines).

While we used all three methods of interpretive synthesis, we found that the line of argument synthesis provided a better conceptualisation of and fit for emerging themes and the complex relationship between the metaphors. The line of argument synthesis conceptualises how experiences of trauma, including interpersonal, systemic, and institutional dimensions of racial oppression, collide with autocratic and monocultural service responses to create ethnically differentiated experiences of mental illness and healthcare. The results of the line of argument synthesis are depicted visually in Fig 2. All of the concepts in the line of argument build upon and relate directly back to first-order accounts. Fig 2 highlights the complex recursive relationship between service access, experience, and outcomes. Below, we discuss the line of argument synthesis highlighting refutational accounts where relevant to our research question. Unless otherwise stated, the literature referenced in this section are papers included in our meta-ethnography and as such constitute the evidence on which these themes are based.

#### Oppression and the medical model of illness

We found that experiences of oppression such as racism impacted help-seeking for symptoms of mental illness across ethnic minority communities. Participant accounts revealed that experiences of racism have multiple interrelated oppressive, social, economic, and traumatic consequences. Experiences of everyday racism [52,54–70], and the trauma such experiences produce, erode mental health, well-being, and capacity and trust for formal help-seeking, particularly when they are replicated in different contexts and over time [56,62,65]. These include experiences of interpersonal verbal and physical racist attacks as well as institutional and societal racism. Participant narratives resonate with the concept of “racial battle fatigue,” which described the cumulative effect of racist micro- and macroaggressions on personal resources [71,72]. As highlighted in previous work [73], our synthesis shows that racist violence does not need to have been experienced personally for it to have an impact on an individual’s mental health and help-seeking for mental illness. Vicarious racism, and the psychological tension associated with living in fear of exposure to racist discrimination and violence (including in healthcare), is particularly strong for established ethnic minority communities who consider racism to be systemic in the UK. For example, historical experiences of slavery and everyday injustice in education and employment characterise experiences of the black community [48,56,57,65,74]. The fear of Islamophobia shapes experiences in Muslim communities [54,63]. For recent migrants, racialisation may be a new experience but they attribute this to negative societal stereotypes and narratives about immigration [67,69]. The indirect effects of racism on formal help-seeking for mental illness are highlighted in narratives describing how prejudice and negative stereotypes produce social [54,57,59,60,66,67,75–82] and economic [59,60,65,66,69,75,79,83] marginalisation. Financial constraints result in low personal resources, creating practical difficulties in accessing mental health support services, including those identified in the metaphor “it’s not possible” (Table 3).

Narratives around social explanations of illness were strong and consistent across the different ethnic minority groups explored. Participants made sense of distress as originating from social risk factors with mental illness seen as a “*social problem*” [49,54,56,63–67,69,74,75,78–80,84–90]. Social stressors were particularly overwhelming for ethnic minority groups who had recently migrated to the UK, particularly in response to civil war and conflict in countries of origin such as refugees and asylum seekers. These included social disruption (i.e., social isolation, unemployment), exposure to previous complex trauma, and dealing with the immigration system described as “*a killer*” and a significant source of anxiety “*It’s like you are on death row waiting to die*” [60,66,75,79,88].

As such, ethnic minority communities perceive help-seeking from a system that cannot begin to accommodate this lived experience to be “useless” (Table 3) [64,66,67,69,74,79,87,89,91].

Participants, including service providers, described how services based on (typically) biomedical/reductionist understandings of ill health deny the impact of wider structural determinants of health, as well as the importance of faith for managing mental illness for religious patients (see metaphor “Epistemic tensions”, Table 3). In addition, some participants perceived that mental health services were epistemologically “white”/“Eurocentric”/”racist” and not designed to meet the needs of people from ethnic minority groups [55–58,71,92–95]. Such experiences contribute to a sense of trauma and marginalisation. Participant accounts resonate with the notion of epistemic injustice [96], a form of hermeneutical injustice related to knowledge including exclusion, silencing, or undervaluing alternative interpretations [48,55–57,59,61,64,67,69,79,87,92]. Consequently, our synthesis showed that many people from ethnic minority groups are searching for alternative ways of getting help and healing compared to what is currently available to them in the way of a strictly western biomedical approach to mental health. While this concern may also apply to the white majority ethnic group, it was considered that the social model of mental illness had (even) more to offer to people where issues of race and culture were central to their lives: “*The race and culture group doesn’t fit in the medical model at all*” [67]. Participants spoke about the need for integrated models of health where there was parity between social and biomedical models of mental illness and between service user and provider voices. This includes better attention to risk factors that have a disproportionate negative impact on the mental health of people from ethnic minority groups such as racism and migration.

#### Vulnerability and social survival in the context of existing marginalisation

As our data show, racism operates, and can directly affect mental health, through the impact of negative stereotypes that present members of a particular ethnic group as inherently problematic and deviant [56,61,62,67,74]. Racism undermines an individual’s sense of personhood and validity and is experienced as traumatising, stigmatising, and disempowering. This includes producing experiences of spoiled/discredited or precarious/discreditable identities [97]. Oppression related to racial identity can result in distress by generating feelings of powerlessness, injustice, and social defeat. In addition to the direct psychological consequences of living with a spoiled or precarious identity, those living with this precarity will take action to preserve or reinforce positive reflections on their identities, and avoid risks of further stigmatisation, which may directly undermine engagement with health services, especially when they are perceived to exacerbate this marginalisation. This is shown by the barriers to acknowledging mental distress identified in the metaphor “not allowed” (Table 3) [48,49,65,67,69,78]. Participants described how oppressive experiences do not allow the space for vulnerability and act as powerful disincentives to falling ill [48]. Consequences include emotional suppression and the development of racialised identities such as the “strong black woman/man” in response to systemic racism and oppression [48,49,65,67,69,78].

Individuals also deny their experiences of mental ill health where this label risks undermining relationships within their existing social network. This includes a reluctance to acknowledge experiences of mental ill health among family members in an effort to prevent spoiling the identities of family members [64,76,78,83,91,95,98–101]. Formal help-seeking becomes synonymous with the acquisition of (additional) stigmatising and oppressive labels and diagnoses [48,81,88,89,102,103], particularly where faith and other communities and families encourage a view that (mental) illness is equated with weakness and moral failings [48,65,78,88,102–105]. A diagnosis of mental illness can therefore be seen as equivalent to a form of social death, which “*cuts short any types of aspirations and hope*” [103], particularly in the context of precarious minority identities, racist social exclusion, and strong interdependence in ethnic minority communities in response to that [49,69,75,78,81,88,89,91,102,103,106,107]. As such, for some families, the need for social survival in the context of social marginalisation results in emotional suppression and the censoring of conversations around mental illness [64,76,78,83,91,98–100,105]. Refutational accounts reveal that there is much heterogeneity between the attitudes of individuals, families, and wider communities in the extent to which mental illness, and formal help-seeking for mental illness, is perceived to be allowed [54,63,78,94,99]. Some participants thought that concerns about stigma and related pressures to suppress emotions and illness were exclusive to older/first generations [89], with evidence of young people finding formal help more acceptable [68,89,94,108]. The intergenerational tensions were revealed in narratives that indicated the presence of secrecy and parental disapproval in relation to service access [99]. For refugee communities, especially unaccompanied minors, there was a particular fear that association with formal “mental” services would result in abandonment by social circle, social isolation, and homelessness [81]. In this context, the word “mental” was experienced as highly stigmatising and this stigma extended to services and diagnoses that used this word.

#### Anticipated fears and costs of service access and provision

The metaphor “not worth taking the risk” (Table 3) reflects concerns that the costs of help-seeking could easily outweigh the opportunities offered by seeking respite from the symptoms of poor mental health. In addition to these anticipated social costs, participants fear that they will not receive safe or equitable care within services themselves. Participants feared that their cultural preferences and beliefs (i.e., spirituality) and social problems would be pathologised as a mental disorder in services [83,85,87,89,95]. Other fears included being misunderstood by service providers in the face of linguistic and conceptual miscommunication (relating to the use of different concepts for illness between patients and doctors) as well as risks of confidentiality breaches [78,98,100], fears of structural racism [57,63,65,67,74,93,109], and medical harm [48,56,58,60,66,69,74,80,106,110]. These fears are related to the perceived lack of cultural competency in services including a lack of diversity in staff teams [48,74].

Our analysis revealed considerable resonance between professionals and lay communities, with ethnic minority staff discussing these fears in the context of their own marginalised identities within services that are perceived to be structurally and epistemologically “white” and “Eurocentric.” Actions by these staff to facilitate culturally or racially sensitive approaches to service delivery are considered too risky to attempt or even discuss within the clinical setting [92,111]. Practitioners also feel silenced when they witness, and attempt to report, racism in diagnostic and treatment decision-making [56,58]. Clinicians describe feeling inhibited in a medical system where it does not feel safe or acceptable to talk about religion or spirituality with their patients without being judged by their colleagues [92]. Fear of rejection from the British medical community for being “anti-modern” or “unscientific” led to the suppression of concerns about the lack of attention to patients’ spiritual and religious needs in services [92]. This related to the epistemological paradigm and culture of mental healthcare in the UK, which was perceived to be reductionist and racist. We found that ethnic minority staff, while more likely to recognise racist practice and respond to the cultural and religious needs of patients, often felt disempowered (within the wider organisation) to intervene. This allows ethnic inequalities in care to perpetuate and indicates that the presence of ethnic minority staff in services is not enough to tackle these. A service provider expressed that it was difficult for her and other ethnic minority staff in general to “fit in” and work in mental healthcare due to the presence of “institutional racism” and the feeling of being “watched and monitored” [95]. The important role of institutional culture in medicine was highlighted by migrant psychiatrists (medically trained outside the UK) who discuss the negative implications of having to adapt their clinical practice to the UK context, from a holistic model that considered faith to a secular model where talk of religion was perceived to be controversial [92]. We did not find any distinct patterns in relation to the use of spiritual explanations for poor mental health across ethnic or faith groups. Interestingly, among those who were not service providers, we identified strong refutational accounts in the extent to which spiritual/religious explanations of illness were perceived positively and/or conflicting with the biomedical paradigm [66,78,85,87,89,94]. Some participants attributed a tendency to view illness through a spiritual lens with low levels of education and mental health literacy [66,94,106]. However, closer inspection revealed a nuanced storyline that indicated a general recognition of the value of holistic illness explanatory frameworks and a desire for institutional cultural change that allowed faith-integrated approaches and better inclusion of, and attention to, religious and spiritual beliefs. These views were shared by communities, service users, carers, and psychiatrists [56,66,74,79,89,92].

Fears and concerns about formal help-seeking resulted in multiple attempts to identify alternative sources of support that would enable individuals to receive mental healthcare without the involvement with mainstream services. Participant narratives revealed that statutory services were commonly viewed, or accessed, as a last resort when “*all the stones have been turned*” and there is “*nowhere else to turn*” [51,56,69,76,89,107].

#### Key junctures in the help-seeking process

The metaphors “turning towards” and “turning away” (Table 3) indicate key junctures in the help-seeking process and highlight experiences that result in engagement and disengagement with services, respectively. It is here that refutational accounts are the strongest. There were refutational narratives relating to turning towards and away from general practitioners (GPs), ethnically similar/dissimilar practitioners [71,93–95,98–101], and informal support [54,64,69,75,76,78,84–87,89,99,103]. In mainstream NHS services, GPs are positioned as the first port of call for help-seeking for mental illness, but most participants do not consider this approach useful. There is a perceived overreliance on medication among GPs, who “*normally just give you tablets*” [78], despite a preference among most participants for talking and trauma informed therapy. That said, some refugee minors appreciated the mood enhancing effects of medication as talking therapy was experienced as (re)traumatising due to its requirement to revisit earlier experiences [81]. Other perceived limitations of GP practice included the short duration of consultations: “*these aren’t 10-minute conversations you can have with someone*!”[89] particularly given the potential need to explain your cultural context and alternative conceptualisations of illness during that time [64,84,85]. Participants also suggest that GPs are “*not qualified enough*” [54] to deal with emotional distress [54,69,78,89,110]. Yet despite these perceptions of low benefit, GP settings were considered to be safer and less stigmatising than psychiatric hospitals [60,69]. Positive experiences with GPs were associated with characteristics of a good therapeutic relationship including approachability and trust “*I just feel that I can talk to her*, *we just start talking about everything and anything*” [69]. Similar storylines emerged from refutational narratives relating to informal support. These accounts revealed the importance of good quality relationships in terms of safety, trust, understanding, and confidentiality [89,108]. The ethnicity of the practitioner did not always appear to be important if the fundamental qualities of a good therapeutic alliance were met [63,67,68,78,99,100]. However, for some participants, having a practitioner from the same ethnic group helped strengthen this therapeutic alliance, overcome linguistic barriers [84,99], and was considered key to recovery [54,64,69,78,85–87,99]. Ethnically dissimilar practitioners were preferred when there were concerns about judgement and confidentiality [98–101]. For example, South Asian GPs were perceived to be cultural gatekeepers of the community and thought to breach patient confidentiality [98,100].

#### Battling against the system

Participants describe having to “*shout to be heard*” within mainstream services [61]. They describe “battling against” a system which was not designed to support them, and which is seen to directly contribute to their sense of victimisation and harm [51,56,60–64,67,69,74,75,77,79–81,84,87,89,99,103,112]. People reflected on the problems of services that ignored the needs and preferences of clients and failed to provide holistic care, while denying the mental health and other consequences of the testimonial injustice, cultural pathologisation, and direct and indirect racist, verbal, physical, and symbolic violence they inflicted. The synthesis showed that these approaches resulted in service users “falling through the net” and feeling like the “victim” of a service unable to support them effectively (Table 3) [51,55,56,61–64,67,69,79,87,88,112]. Such experiences exacerbated people’s mental distress and their sense of systemic injustice and, for some, led to a “turning away” from the service entirely.

The persistence of “business as usual” in services, despite a plethora of existing evidence highlighting the problems and documenting community solutions and recommendations to improve care, was a source of frustration raised by service providers and people from ethnic minority groups [59,79,87,95]. The lack of progress despite numerous policies and recommendations was attributed to superficial attempts at coproduction and insufficient adoption of community solutions by services. Service providers suggested that community solutions were considered too radical for statutory services and commissioners to implement. In addition, service providers thought that the implementation of policies was discriminatory and done by seniors from “privileged middle class white male backgrounds” who were perceived to have little understanding of the needs of people from ethnic minority groups [95]. Failures in coproduction and implementation of solutions exacerbated feelings of mistrust and created a sense of hopelessness. This was felt particularly strongly by people from African Caribbean communities where inequalities are considered most entrenched and where there have been multiple unsuccessful attempts to address them.

#### Business as usual is not enough

As shown in Table 3 (metaphor “business as usual is not enough”), we found a wide array of solutions and recommendations in the literature. Table 4 presents a list of the key desired solutions and recommendations by each metaphor category relating to service access, experience, and outcomes. A major recommendation related to the need for a paradigm shift from traditional western “Eurocentric” and “medical” models of care to more democratic, holistic, and person-centred approaches [52,54,56,57,64,66–69,74,75,79,81,83,85,87–89,92,110–113]. Studies show that services and medical training should move away from a medical “symptoms and labels” approach to a “whole-person” understanding of distress [56]. The third sector was identified as a source of holistic and nonstigmatising support and studies highlighted the need for better collaboration between statutory services, the third sector, and existing community assets. This was perceived to require the provision of financial and political support to third sector organisations and collaborative approaches that allowed statutory and community services to meet in the middle. Other suggested solutions included the removal of stigmatising language such as “mental” or “depression” from services, and better access to talking therapy, creative therapies, and well-being activities. Interestingly, while we found very few accounts relating to recovery and healing, these were all largely related to creative activities (see metaphor “turning towards”, Table 3). Creative engagement and expression of illness narratives was viewed as empowering, healing, and transformative [64,65,75,84,87,112]. This included storytelling and sharing of illness narratives, joining a singing group “release my feelings” and “heal my heart” [84], and journaling. Participation in qualitative research allowed participants to share their story and be heard, sometimes for the first time, and was experienced as therapeutic. In this sense, engaging with and making sense of the illness narrative through creative expression helped transform the illness experience (empowering/normalising/humanising). Stories were seen to allow and open the door to healing “*I didn’t feel like an alien*, *it didn’t feel like an illness or a sick thing or a broken thing*. *I could see myself as a person who has gone through a lot and is reacting actually quite appropriately to the distress that has been heaped on her… So that was actually the turning point in my life*” [64].

**Table 4:** Desired solutions and changes to improve access, experience, and outcomes.

Metaphor categories relating to access, experiences, and outcomes	Summary of key arguments	Key desired solutions and changes to mental health services
Practical barriers in help-seeking or help-giving, which relate to the shortage of resources	Gaps within services (time to ask patients about religious/cultural needs; low availability of interpreters). Economic constraints in socially disadvantaged communities such as limited personal and financial resources to identify help, services (i.e., in absence of a computer), and alternative treatments.	• Tackle economic disadvantage and racial discrimination in employment sector. • Create mental health and well-being centres with open access in the local communities staffed by people from those communities • Increase availability of interpreters to cover diverse range of languages particularly in inpatient settings • Remove financial barriers to well-being activities (including access to quality green space)
Fears and concerns about service access and provision that relate to anticipated costs and benefits	Experiences of systemic racism result in loss of trust and reluctance to disclose personal difficulties including vulnerability and illness. Fears about costs include exposure to further oppressive experiences and consequent marginalisation, misunderstandings and misdiagnosis, and medical harm. Benefits of accessing statutory services perceived to be low due to lack of focus on structural causes of illness, services perceived to be epistemologically “white/Eurocentric,” overreliance on medication, and low perceived availability of holistic care.	• Remove stigmatising language such as “mental” and “depression” from services • Ensure coproduction of services that are meaningful to BME communities including culturally appropriate training • Implement community and workplace advocacy and individual empowerment of people from ethnic minority groups • Address institutional racism with clear commitments and evidence of system change and improved outcomes • Develop partnerships with community organisations across sectors to bring about service change.
Experiences, consequences, and outcomes of service use	Services experienced as autocratic, monocultural “white/Eurocentric,” oppressive, reductionist, medication-focused, violent, traumatising, and racially discriminatory. Refutational accounts relating to positive and negative experiences indicate need for safe, nonstigmatising, inclusive spaces to be seen, heard, and understood without judgement or bias. Lack of progress in reducing ethnic inequalities in mental health attributed to lack of commitment, institutional racism, and absence of authentic coproduction.	• Need for a whole-person, whole system approach including holistic person-centred care responsive to social and structural issues and allows attention to faith and spirituality • Earlier and improved access to culturally appropriate talking therapy and well-being activities. • Strategies to tackle ethnic inequalities in mental healthcare should include a commitment to make services less aversive, more consensual, changing the culture of care and measurable improvements in experience and outcomes • Services to commit to being anti-racist organisations • Provide support to help patients address symptoms associated with medication such as weight gain and reduced capacity for physical activity • Increase representation of ethnic minority staff in leadership and senior management

BME, black and minority ethnic.

In addition, studies highlighted a need for community and workplace interventions to tackle forms of societal oppression and enable empowerment of people from ethnic minority groups, particularly the black community where the impact of systemic racism was considered to be deeply ingrained [56,57,59,64,65,74,88]. Studies highlighted that strategies to tackle ethnic inequalities in mental healthcare need to go beyond increasing recruitment of ethnic minority staff and must include empowerment of existing staff and the introduction of anti-racist approaches to tackle racist practice and cultural misunderstandings in clinical care [56,114]. This includes building capacity of service providers to deliver culturally resonant and anti-racist treatment and care through training [55,56,60,77,81,82,88,92,98,111].

## Discussion

This synthesis found that a combination of individual, systemic, and structural factors act to create and sustain ethnic inequalities in mental healthcare. Based on the qualitative studies included in our review, we found that people from diverse ethnic minority backgrounds had similar negative perceptions and experiences of mental healthcare. A key overarching theme related to the presence of significant fear and concern about the suitability of the current model of care to deliver useful, safe, and equitable mental healthcare to ethnically diverse communities. This mainly relates to the perceived dominance of a biomedical and “white” colonial epistemology within services. Current methods of assessment and treatment, in psychiatry in particular, are experienced as reductionist, oppressive, and racist. A major criticism related to the inability of services to consider the whole person and the lived experience of people from ethnic minority communities during assessment and treatment. This lived experience was diverse across the 14 ethnic groups but frequently included an important role for religion/spirituality in mental health and experiences of racism, migration, fragile social circumstances, complex trauma, social marginalisation, family and community interdependency, and English language proficiency. In this sense, the need to be seen, heard, and understood without judgement or bias and receive appropriate treatment was a strong theme across ethnic groups and one that also applies to the majority ethnic group. The synthesis found that the most powerful intersections related to experiences of oppression and these experiences were perceived to be most systemic and deeply entrenched for black ethnic groups. Our findings suggest that oppressive experiences are often created and reenacted in services especially when a narrow “symptom and label” approach is taken during clinical assessment and treatment planning. This approach is experienced as a form of epistemic injustice because it excludes wider causes of mental illness such as racism and migration stress and locates the pathology in the individual rather than broader social circumstances. Negative service experiences and outcomes perpetuate fears about service use in the community, especially when these replicate societal experiences of marginalisation and systemic oppression. Consequences include service avoidance and disengagement, and a reliance on nonstatutory support services, mostly located in the third sector.

We found that service recommendations to improve experiences of people from ethnic minority groups are well documented in the literature. The lack of progress in tackling inequalities despite the existence of numerous policies and recommendations is attributed to superficial and discriminatory attempts at coproduction and insufficient adoption of community solutions by statutory services. Policy implementers and key decision makers are perceived to come from white privileged backgrounds and to have little understanding of the needs of people from ethnic minority groups. Our synthesis indicates pervasive challenges across primary and secondary care settings including adult and child services.

Previous reviews that have included experiences of ethnic minority groups have mostly focused on individual-level barriers to access and/or experiences of a heterogenous mix of marginalised social groups, or specific ethnic groups such as South Asians [56,115–117]. One review explored the experiences of people from marginalised social groups classified as “hard to reach” and highlighted the need for socially conscious models of mental healthcare to improve access to primary care [115]. A recent rapid review of qualitative and quantitative evidence generated in the past ten years found that ethnic inequalities in healthcare, including mental health, are rooted in racism [32]. Our synthesis shows that individuals and communities who have experienced multiple layers of oppression, particularly related to ethnic/racial identity, require models of mental healthcare that are explicitly empowering and anti-oppressive. This requires services to tackle obstacles to person-centred care and acknowledge the wider impact of structural inequalities in methods of assessment and treatment including intergenerational racial trauma. While the importance of person-centred care in psychiatry is universally recognised, like others [118,119], our findings highlight several institutional barriers to the provision of person-centred care and show how these have a disproportionate negative impact on people from ethnic minority groups. This includes the presence of autocratic clinical encounters, racial stereotypes, fear and uncertainty among clinicians in calling out racist/culturally inappropriate practice, and lack of time and resources to accommodate patients’ language, spiritual, and religious needs. These barriers to person-centred care were seen to reflect the current structure of mental healthcare, which was perceived to be inadequate to attend to individual patient needs, particularly the need for holistic care. While having a disproportionate negative impact on ethnic minority groups, our findings indicate potential challenges for all service users including from the majority ethnic group. In this context, we found that intersections relating to experiences of racism, migration, fragile social circumstances, complex trauma, social marginalisation, the important role of religion in a person’s life, and English language proficiency were more conceptually important and useful than crude ethnic group classifications. A recent quantitative study exploring mental health service use in men also found important intersections between social deprivation, religion, and ethnicity [120].

While we did not find many accounts of recovery, participants associated it with emotional release and empowerment. We found common themes associated with finding validation and transformation through expression of trauma in safe spaces. Empowering activities included finding solidarity and validation in anti-racist spaces in the third sector. Emotional release was associated with creative expression of emotions such as singing, journaling, and sharing of personal illness narratives. While art therapy has been identified as an anti-oppressive practice, there is sparse research exploring the effectiveness of this approach for people from ethnic minority groups in the UK. This approach may be particularly useful for people who have experienced complex trauma and where nonverbal approaches may be more accessible and acceptable (in our study, this included young refugees and recent migrants) [121]. Nature was also identified as a nonstigmatising and healing space for mental ill health. A recent review highlighted the potential for green space to progress health equity [122]. However, this would require significant efforts to tackle ethnic inequalities in access to good quality and safe green space [123]. Our findings highlight the need for better inclusion, integration, and accessibility of nature and creative therapy as treatment options in services and more research exploring the effectiveness of art-integrated approaches for people from ethnic minority groups. Social prescribing [124] offers a holistic approach to health and well-being; however, we did not find any articles exploring experiences of this approach in ethnic minority groups for mental health. A recent systematic review exploring social prescribing in migrants found that it was associated with increased empowerment and self-esteem [125]. However, most of the studies were of low quality and there is a need for robust evaluations of social prescribing for ethnic minority groups particularly in relation to mental health and well-being [125].

Our findings extend previous work in several ways. Firstly, by highlighting important recursive relationships between service access, experience, and outcomes and across primary, secondary, and third sector settings. Secondly, by bringing together the experiences of all studied ethnic minority groups and showing how intersections such as religion, oppression related to race and migrant status, and experiences of complex trauma create ethnically differentiated experiences. Thirdly, the inclusion of service provider narratives from a range of statutory and nonstatutory settings provided important triangulation and a deeper understanding of the fears and concerns raised by service users, carers, and communities. This includes understanding structural and institutional barriers to improving cultural competence and eradicating racism within services. The involvement of a service user, carer, and provider advisory group provided some insight into the extent which identified challenges from the literature were still considered to be relevant. This was important given the wide timeline of our data. These discussions revealed that service providers from a range of ethnic and social backgrounds thought that they were working in structurally oppressive services described often in our meetings as a “broken system.” This wide epistemological lens has helped shed light on existing epidemiological data highlighting persistent ethnic variations in utilisation of mental health services. This includes understanding why all ethnic minority groups have disparate patterns of service utilisation [15], including low primary care access and higher rates of compulsory pathways to mental healthcare compared to the majority ethnic group [7].

Strengths of the synthesis include inclusion of experiences of 14 diverse ethnic minority groups, from 15 cities across the UK, and covering a period of just over two decades. We provide an understanding of the barriers related to tackling cultural pathologisation, racist practice, and attending to the spiritual and religious needs of patients within clinical settings. The exclusion of literature from outside the UK was important to contextualise these experiences, particularly in terms of understanding inequalities in the context of free healthcare, established equality legislation, and significant research and policy efforts to understand and tackle ethnic inequalities in mental healthcare. Our findings indicating the need for decolonised and destigmatising approaches to mental healthcare have potential relevance to healthcare settings across the Global North and South. Limitations of included literature include the low number of studies that took a multisectoral approach to research (8%). As we found during our synthesis, multisectoral approaches can prevent problematic epistemological imbalances in the creation of knowledge in this field where the third sector plays a significant role in supporting ethnic minority communities (and often has more cultural expertise than academics and statutory services). Other limitations relate to the lack of reporting of generational status of participants. In general, we found very low recording of social identities related to class and generational status in primary studies. This prevented exploration of important intersectional differences relating to these characteristics [126]. A large proportion of primary studies focused on the experiences of BME/black/South Asian populations. These were often crudely clustered by ethnic minority status, skin colour, or continent and made it difficult to get a sense of nuances by individual ethnic group. Missing or understudied ethnic groups included those with “Mixed,” Bangladeshi, Bengali, Sri Lankan, Arab, and white minority ethnicity including white Gypsy/Travellers. The large volume of data may have affected the depth of the analysis. The presence of negligible differences across studies/ethnic groups and resonance with our diverse lived experience advisory group provided some reassurance against this. While we found considerable resonance within our multidisciplinary advisory group practitioners, experiences may vary by geographic area and clinical setting. The number of studies exploring the views and perceptions of service providers was low compared to service users. Service user experiences may also differ by ethnic diversity of service providers by geographic area, clinical setting, and over time. While we did not explore this formally, we did not get a sense of trends by time or geographic location. This may be because criticisms largely relate to the organisation of mental health services and oppressive processes of psychiatric assessment and treatment rather than individual practitioners.

The academic study team represent the following interdisciplinary backgrounds: anthropology (AM), epidemiology of ethnic inequalities in health including mental health (NB), sociology (SK, RC), primary care mental health (CCG), and psychiatry (SS). In addition, NB has experience of raising awareness of mental health in ethnic minority communities including signposting to statutory and nonstatutory services. Backgrounds of the advisory group members included lived service user and carer experience, mental health social work, clinical psychology, and representation from a specialist mental health third sector organisation providing support to people from ethnic minority communities. The academic and advisory team included a mix of intersections relating to age, gender, and ethnicity. This provided a wide epistemological lens in terms of understanding ethnic inequalities in access, experiences, and outcomes. There was considerable heterogeneity within the research team in the type of analytical approach utilised during data extraction and analysis. NB liked to see and understand the overarching storyline and wider explanations (resulting in larger data extraction from the whole paper, mapping of storylines, and attention to the “starting point” of the story for each cluster/ethnic group). RC took the most critical approach in identifying how experiences were differentiated by ethnicity, reflecting on the extent to which experiences could be common to the majority ethnic group and characteristic of mental health services in general. AM focused on key words and phrases and paid attention to the way narratives were expressed and articulated. The funder did not influence the meta-ethnography analysis. The reviewers have no conflicts of interest.

Our synthesis indicates a need for better alignment of statutory mental health services with the lived experience of ethnic minority communities. These epistemic shifts require authentic engagement with ethnic minority communities in the development and evaluation of clinical policies, training, and services that go beyond superficial methods of coproduction [114]. Perhaps most importantly, strategies to tackle ethnic inequalities in mental healthcare require an evaluation of individual, systemic, and structural obstacles to authentic and meaningful coproduction and implementation of existing research and community recommendations in services.

The powerful role of systemic racism as a risk factor for illness for those in ethnic minority groups, and the presence of racist practice in services, calls for implementation of explicitly anti-oppressive and anti-racist approaches to mental healthcare and a clear demonstration to ethnic minority communities of service improvement. This requires commissioners to consider a wider range of nonstigmatising holistic services centred around well-being and empowerment. Examples include better access to treatment approaches that help create safe nonstigmatising spaces for emotional expression such as art-based and trauma-informed therapy. For clinicians, this means better awareness and sensitivity to how experiences of poor listening and engagement, and exclusion of social risk factors during assessment, can replicate wider experiences of marginalisation and oppression. As suggested by others [114,126], true equity in mental healthcare requires a shift from a focus on individual symptoms to better attention to structural causes of inequality. This approach offers a more empowering and safer model of care for people who are disproportionately affected by social oppression. This includes marginalised social groups from the majority ethnic group. We recommend that clinical teams and practitioners review current approaches to assessment and treatment through an anti-oppressive lens. This includes awareness of how use of stigmatising language such as clinical diagnoses can contribute to ethnic inequity in access, uptake of treatment, and recovery.

Further research should take a mixed methods and lived experience approach to assess and evaluate processes of service coproduction and change. Intersections related to experiences of racism, migration, religion, complex trauma, and English language proficiency might be more useful than crude ethnic group classifications both in mental healthcare and research. Finally, there is a need for high-quality research exploring experiences of ethnic minority groups by intersections of class and generational status. This multipronged approach offers a more promising opportunity for the eradication of ethnic inequalities in service engagement and more effective treatment of mental ill health in ethnic minority groups.

## Supporting information

S1 PRISMA Checklist PRISMA checklist. (DOCX)

S1 Appendix Search terms by database. * Indicates a wildcard search that retrieves variations of words that start with the same letters.
(DOCX)

S2 Appendix List of articles included in the review. *Where participants are service providers, this relates to the ethnic group under discussion NR, Not reported by primary study authors.
(DOCX)

## Abbreviations

AFC: African Caribbean
BME: black and minority ethnic
CASP: Critical Appraisal Skills Programme
GP: general practitioner
PPPA: patient, public, and practitioner advisory
QES: Qualitative Evidence Synthesis
STARLITE: Standards for Reporting Literature searches
UK: United Kingdom
