# Supplementary material for: Understanding ethnic inequalities in mental healthcare in the UK: A meta-ethnography
Source: PLoS Med. 2022 Dec 13;19(12):e1004139. doi: 10.1371/journal.pmed.1004139 (PMC9746991; doi:10.1371/journal.pmed.1004139)
Supplement: S1 Appendix — * Indicates a wildcard search that retrieves variations of words that start with the same letters. (DOCX) [file pmed.1004139.s002.docx]

Appendix 1: Search terms by database

| Category | PsycINFO | Medline | CINAHL | | Social care online |
| --- | --- | --- | --- | --- | --- |
| Ethnic minority group | ethnic* OR BME OR BAME OR cultur* OR multicultur* OR transcultur* OR religi* OR Black OR African OR African Caribbean OR Sudanese OR Somali OR Ghanaian OR Nigerian OR White OR Asian OR Chinese OR Indian OR Bangladeshi OR Pakistani OR Punjabi OR Gujarati OR Bengali OR Arab* OR Sri Lankan OR Dual Heritage OR Iranian OR Eastern Europe* OR Polish OR Irish OR Turkish OR Roma* OR English OR Scottish OR Welsh OR British OR Greek OR Albanian OR Gyps* OR Traveller* OR Muslim OR Sikh OR Hindu OR Jewish OR Christian OR Buddhist OR Jain OR Migrant* OR immigra* OR refugee* OR asylum  OR exp refugees  OR exp "Racial and Ethnic Differences"/ or exp Cultural Sensitivity/ or exp Cross Cultural Differences/ or exp Diversity/ or exp "Racial and Ethnic Groups"/ | ethnic* OR BME OR BAME OR cultur* OR multicultur* OR transcultur* OR religi* OR Black OR African OR African Caribbean OR Sudanese OR Somali OR Ghanaian OR Nigerian OR White OR Asian OR Chinese OR Indian OR Bangladeshi OR Pakistani OR Punjabi OR Gujarati OR Bengali OR Arab* OR Sri Lankan OR Dual Heritage OR Iranian OR Eastern Europe* OR Polish OR Irish OR Turkish OR Roma* OR English OR Scottish OR Welsh OR British OR Greek OR Albanian OR Gyps* OR Traveller* OR Muslim OR Sikh OR Hindu OR Jewish OR Christian OR Buddhist OR Jain OR Migrant* OR immigra* OR refugee* OR asylum  OR cross-cultural comparison/ or cultural diversity/ | AB ethnic* OR BME OR BAME OR cultur* OR multicultur* OR transcultur* OR religi* OR Black OR African OR African Caribbean OR Sudanese OR Somali OR Ghanaian OR Nigerian OR White OR Asian OR Chinese OR Indian OR Bangladeshi OR Pakistani OR Punjabi OR Gujarati OR Bengali OR Arab* OR Sri Lankan OR Dual Heritage OR Iranian OR Eastern Europe* OR Polish OR Irish OR Turkish OR Roma* OR English OR Scottish OR Welsh OR British OR Greek OR Albanian OR Gyps* OR Traveller* OR Muslim OR Sikh OR Hindu OR Jewish OR Christian OR Buddhist OR Jain OR Migrant* OR immigra* OR refugee* OR asylum  OR AB ethnicity or race or culture or minority or minorities OR TI ethnicity or race or culture or minority or minorities | TI ethnic* OR BME OR BAME OR cultur* OR multicultur* OR transcultur* OR religi* OR Black OR African OR African Caribbean OR Sudanese OR Somali OR Ghanaian OR Nigerian OR White OR Asian OR Chinese OR Indian OR Bangladeshi OR Pakistani OR Punjabi OR Gujarati OR Bengali OR Arab* OR Sri Lankan OR Dual Heritage OR Iranian OR Eastern Europe* OR Polish OR Irish OR Turkish OR Roma* OR English OR Scottish OR Welsh OR British OR Greek OR Albanian OR Gyps* OR Traveller* OR Muslim OR Sikh OR Hindu OR Jewish OR Christian OR Buddhist OR Jain OR Migrant* OR immigra* OR refugee* OR asylum | ethnic* OR BME OR BAME OR cultur* OR multicultur* OR transcultur* OR religi*  OR Black OR African OR African Caribbean OR Sudanese OR Somali OR Ghanaian OR Nigerian OR White OR Asian OR Chinese OR Indian OR Bangladeshi OR Pakistani OR Punjabi OR Gujarati OR Bengali OR Arab* OR Sri Lankan OR Dual Heritage OR Iranian OR Eastern Europe* OR Polish OR Irish OR Turkish OR Roma* OR English OR Scottish OR Welsh OR British OR Greek OR Albanian OR Gyps* OR Traveller* OR Muslim OR Sikh OR Hindu OR Jewish OR Christian OR Buddhist OR Jain OR Migrant* OR immigra* OR refugee* OR asylum |
| Mental ill-health | mental OR distress* OR psycholog* OR psychiat* OR depression OR anxiety OR psychosis  OR exp Mental Disorders/ OR exp Mental Health/ OR exp Psychological Stress/ or exp Stress/ OR exp Mental Health Services/ OR exp trauma/ OR mood OR abuse OR neglect | mental OR distress* OR psycholog* OR psychiat* OR depression OR anxiety OR psychosis  OR exp Mental Disorders/ OR Mental Health/ OR exp Stress, Psychological/ OR exp Mental Health Services/ | AB mental health services or mental health care OR AB mental health or mental illness or mental disorder or psychiatric illness OR AB mental OR distress* OR psycholog* OR psychiat* OR depression OR anxiety OR psychosis | TI mental health services or mental health care OR TI mental health or mental illness or mental disorder or psychiatric illness OR TI mental OR distress* OR psycholog* OR psychiat* OR depression OR anxiety OR psychosis | Mental disorder or mental health care or psychological distress or mental OR distress* OR psycholog* OR psychiat* OR depression OR anxiety OR psychosis |
| Help-seeking | exp Health Care Utilization/ or exp Help Seeking Behavior/ OR social prescribing OR sign-posting OR self-referral/ | "Patient Acceptance of Health Care"/ OR help-seeking.mp. OR social prescribing.mp. OR signposting.mp. OR self referral.mp. | AB help-seeking OR AB social prescribing OR AB signposting OR AB self referral OR TI help-seeking OR TI social prescribing OR TI signposting OR TI self referral | | Help-seeking or “help seeking” or utilisation or “social prescribing” or “self referral” or signposting |
| Experience | experience* or belief* or perception* or view* or attitude* or perspective* | experience* or belief* or perception* or view* or attitude* or perspective* | AB (experience* or belief* or perception* or view* or attitude* or perspective*) OR TI (experience* or belief* or perception* or view* or attitude* or perspective*) | | experience* or belief* or perception* or view* or attitude* or perspective* |

BME/BAME: Black and minority ethnic

***** Indicates a wildcard search that retrieves variations of words that start with the same letter
