# Supplementary material for: Understanding ethnic inequalities in mental healthcare in the UK: A meta-ethnography
Source: PLoS Med. 2022 Dec 13;19(12):e1004139. doi: 10.1371/journal.pmed.1004139 (PMC9746991; doi:10.1371/journal.pmed.1004139)
Supplement: S2 Appendix — *Where participants are service providers, this relates to the ethnic group under discussion NR, Not reported by primary study authors. (DOCX) [file pmed.1004139.s003.docx]

Appendix 2: List of articles included in the review

| **Reference** | **Publication year** | **Sample setting** | **Stakeholder group** | **Sample (n)** | **Ethnicity studied*** | **Age** | **gender** | **Data collection method** |
| --- | --- | --- | --- | --- | --- | --- | --- | --- |
| Ally et al. [1] | 2015 | Statutory secondary adult | Carer | 4 | East African Muslims | 22 to 40 | Female & male | Interview |
| Bhardwaj [2] | 2001 | Community | Community, service user & statutory & non-statutory provider | NR | South Asian | 14 to 30 | Female | Interview & focus groups |
| Birch et al. [3] | 2020 | Community | Community | 24 | BME | 17 to 27 | Female & male | Interview & art workshops |
| Burr [4] | 2002 | Statutory primary & secondary adult | Service provider | 29 | BME | NR | Female & male | Interview & focus groups |
| Burr et al. [5] | 2004 | Community | Community | 46 | South Asian | 21 to 61 | Female | Interview & focus groups |
| Campbell et al. [6] | 2004 | Community & services | Statutory, voluntary, user & lay community | 30 | AFC | NR | Female & male | Interview & focus groups |
| Century et al. [7] | 2007 | Statutory primary care | Service providers | 13 | Refugee | NR | Female & male | Interview |
| Chakraborty et al. [8] | 2009 | Statutory secondary adult | Service users | 20 | Black Caribbean & white British | 26 to 64 | Female & male | Interview |
| Chandler-Oatts et al.[9] | 2008 | Statutory secondary adult | Service users & providers | 33 | AFC | NR | Female & male | Focus groups |
| Chowbey et al. [10] | 2012 | Community & service | Relatives/carers, community, statutory & non-statutory service providers | 42 | BME | NR | Female & male | Interview & focus groups |
| Chtereva et al. [11] | 2017 | Community | Community | 16 | Central/Eastern European | 20 to 56 | Female & male | Interview |
| Cinnirella et al. [12] | 1999 | Community | Community | 52 | BME | NR | Female | Interview |
| Dura-Vila et al. [13] | 2011 | Statutory secondary adult | Service providers | 20 | BME | late 20's to 50's | Female & male | Interview |
| Edge et al. [14] | 2005 | Community | Community & service users | 12 | Black Caribbean | 19 to 43 | Female | Interview |
| Edge [15] | 2008 | Community antenatal clinics | Community | 12 | Black Caribbean | 20 to 42 | Female | Interview |
| Edge et al. [16] | 2010 | Community antenatal clinics | Community & service users | 12 | Black Caribbean | 18 to 43 | Female | Interview |
| Edge [17] | 2010 | Statutory primary and secondary & non-statutory | Service providers | 42 | BME | NR | Female & male | Interview & focus groups |
| Faulkner [18] | 2014 | Community | Service users | 40 | BME | NR | Female & male | Focus groups |
| Fish et al. [19] | 2018 | Non-statutory | Service users & providers | 17 | Refugee & asylum seekers | NR | Female & male | Focus groups |
| Green et al. [20] | 2002 | Statutory primary and secondary & non-statutory | Service users | 42 | Chinese | 29 to 60 | Female | Interview |
| Green et al. [21] | 2006 | Statutory primary & non-statutory | Service users | 42 | Chinese | 29 to 60 | Female | Interview |
| Gunputh [22] | 2015 | Community | Community | 9 | South Asian | 13 to 19 | Female & male | Interview |
| Gurpinar-Morgan et al. [23] | 2014 | Statutory secondary child | Service users | 5 | BME | 16 to 18 | Female & male | Interview |
| Hills et al. [24] | 2013 | Statutory and non-statutory | Service users & service providers | 78 | African | NR | Female & male | interview |
| Hussain et al. [25] | 2002 | Statutory secondary adult | Service users | 13 | South Asian | NR | Female | interview |
| Jim et al. [26] | 2007 | Community | Service users | 8 | Chinese | 19 to 37 | Female & male | interview |
| Kai et al. [27] | 1999 | Community | Community | 104 | Pakistani and Bangladeshi | 16 to 65 | Female & male | interview |
| Kalathil et al. [28] | 2011 | Community | Service users | 27 | African/AFC/  South Asian | 25 to 65 | Female | Interview |
| Kang et al. [29] | 2020 | Statutory secondary adult | Service providers | 9 | BME | NR | Female & male | interview |
| Keating et al. [30] | 2002 | Statutory secondary adult | Service users, carers & providers | 191 | African/AFC | NR | Female & male | Focus groups |
| Keating et al. [31] | 2004 | Statutory secondary adult | Service users, carers & providers | 81 | African/AFC | 20 to 74 | Female & male | Interview & focus groups |
| Klineberg et al. [32] | 2013 | Community | Community | 30 | BME | 15 to 16 | Female & male | interview |
| Li et al. [33] | 2014 | Community | Community | 8 | Chinese | 61 to 92 | Female & male | interview |
| Mahmood [34] | 2012 | Community | Community | 7 | Pakistani | 21 to 35 | Male | Interview |
| Majumder et al. [35] | 2015 | Statutory secondary child | Service users | 15 | Refugee | 15 to 18 | Female & male | Interview |
| Majumder et al. [36] | 2019 | Statutory secondary child | Service users | 15 | Refugee | 15 to 18 | Female & male | Interview |
| Majumder et al. [37] | 2019 | Statutory secondary child | Service users | 30 | Refugee | 15 to 18 | Female & male | interview |
| Mallinson et al. [38] | 2007 | Statutory primary care | Service users | 58 | Pakistani | 19 to 65 | Female & male | Interview |
| Mantovani et al. [39] | 2017 | Community | Community | 26 | African/AFC | 24 to 75 | Female & male | Interview |
| Marshall et al. [40] | 1999 | Statutory primary and secondary & non-statutory | Service users & service providers | 15 | South Asian | 16 to 28 | Female | Interview |
| McEvoy et al. [41] | 2017 | Community & service | Community & service providers | 8 | Jewish | NR | Female & male | Interview |
| Mclean et al. [42] | 2003 | Community & service | Service users, carers, community, statutory & non-statutory providers | 30 | AFC | NR | Female & male | Interview & focus groups |
| Bhui et al. [43] | 2002 | Community | Service users & community | 116 | BME | 25 to 50 | Female & male | interview |
| Palmer et al. [44] | 2007 | Community | Service users & community | 9 | Somali refugee | 18 to 62 | Female & male | interview |
| Patel [45] | 2018 | Community | Community | 9 | Gujarati | 24 to 65 | Female & male | interview |
| Rabiee et al. [46] | 2013 | Statutory secondary adult | Service users, carers, statutory & non-statutory providers | 97 | African/AFC | NR | Female & male | Interview & focus groups |
| Rae [47] | 2014 | Community | Community | 12 | Somali refugee | 30 to 59 | Male | Interview & focus groups |
| Reavey et al. [48] | 2006 | Statutory primary and secondary & non-statutory | Service providers | 37 | South Asian | NR | Female & male | Interview & focus groups |
| Robinson et al. [49] | 2011 | Community | Community | NR | BME | 18 to 55 | Male | Focus groups |
| Selkirk et al. [50] | 2012 | Community | Community | 9 | Polish | 28 to 40 | Female & male | Interview |
| Shefer et al. [51] | 2013 | Community | Service users & community | 103 | BME | 22 to 69 | Female & male | Focus groups |
| Tuffour et al. [52] | 2019 | Statutory secondary adult | Service users | 12 | African | 19 to 44 | Female & male | Interview |
| Upadhya [53] | 2015 | Community | Community | 5 | Nepalese | 19 to 29 | Female & male | Interview |
| Vincent et al. [54] | 2013 | Statutory secondary adult | Service users | 7 | Refugee & asylum seekers | 19 to 42 | Female & male | Interview |
| Wagstaff et al. [55] | 2018 | Statutory secondary adult | Service users | 7 | Black | 31 to 64 | Male | Interview |
| Weatherhead et al. [56] | 2010 | Community | Community | 14 | South Asian | 20+ | Female & male | Interview |
| Yeung et al. [57] | 2017 | Non-statutory | Service users & families | 32 | Chinese | 25 to 64 | Female & male | Interview |
| Bailey et al. [58] | 2021 | Community | Community | 8 | Black Caribbean | 65 to 79 | Female & male | Interview |
| Hussain et al. [59] | 2021 | Community | Community & service provider | 8 | Pakistani | 30 to 50 | Female & male | Interview |
| Lawrence et al. [60] | 2021 | Statutory secondary adult | Service users | 35 | Black Caribbean, White British, White Other | 21 to 50 | Female & male | Interview |
| Meechan et al. [61] | 2021 | Community | Community | 10 | Black | 16 to 18 | Male | Interview |
| Olaniyan et al. [62] | 2022 | Community | Community | 48 | Black African/AFC, South Asian | NR | Female & male | Interview |
| Pilav et al. [63] | 2022 | Perinatal mental health service | Service users | 18 | BME | 18 to 46 | Female | Interview |
| Dare et al. [64] | 2022 | Community | Service users, service providers, community | 6 | Black African | 22 to 55 | Female & male | Interview |
| Lawrence et al. [65] | 2021 | Statutory secondary adult | Service users | 35 | Black Caribbean and White | 21 to 50 | Female & male | Interview |
| Simkhada et al. [66] | 2021 | Community & statutory primary | Community, service user & service provider | 21 | Nepali and Iranian | NR | Female & male | Interview |

*Where participants are service providers, this relates to the ethnic group under discussion

NR: Not reported by primary study authors, BME: Black and minority ethnic, AFC: African Caribbean

References

1. Ally F, Brennan T. Schizophrenia, psychiatry and East African Muslim families in the United Kingdom: a pilot study. Mental Health & Social Inclusion. 2015;19(1):45-51.

2. Bhardwaj A. Growing up young, Asian and female in Britain: A report on self-harm and suicide. Feminist review. 2001;68(1):52-67.

3. Birch J, Rishbeth C, Payne SR. Nature doesn't judge you - how urban nature supports young people's mental health and wellbeing in a diverse UK city. Health & Place. 2020;62:102296.

4. Burr J. Cultural stereotypes of women from South Asian communities: mental health care professionals' explanations for patterns of suicide and depression. Soc Sci Med. 2002;55(5):835-45.

5. Burr J, Chapman T. Contextualising experiences of depression in women from South Asian communities: a discursive approach. Sociol Health Illn. 2004;26(4):433-52.

6. Campbell C, Cornish F, McLean C. Social Capital, Participation and the Perpetuation of Health Inequalities: Obstacles to African-Caribbean Participation in 'Partnerships' to Improve Mental Health. Ethnicity & Health. Vol.9(4), 2004, pp. 313-335.

7. Century G, Leavey G, Payne H. The experience of working with refugees: Counsellors in primary care. British Journal of Guidance & Counselling. Vol.35(1), 2007, pp. 23-40.; 2007.

8. Chakraborty AT, McKenzie K, King M. Discrimination, ethnicity and psychosis-A qualitative study. Ethnicity and Inequalities in Health and Social Care. Vol.2(1), 2009, pp. 18-29.

9. Chandler-Oatts J, Nelstrop L. Listening to the voices of African-Caribbean mental health service users to develop guideline recommendations on managing violent behaviour. Diversity in Health and Social Care. 2008;5(1):31-41.

10. Chowbey P, Salway S, Ismail M. Influences on diagnosis and treatment of eating disorders among minority ethnic people in the UK. Journal of Public Mental Health. 2012;11(2):54-64.

11. Chtereva E, Ward T, Ramsey-Wade C. Becoming 'another brick in the wall': A thematic analysis of Central and Eastern European immigrants' experience of psychological distress and help-seeking. Counselling Psychology Review. Vol.32(3), 2017, pp. 26-38.; 2017.

12. Cinnirella M, Loewenthal KM. Religious and ethnic group influences on beliefs about mental illness: a qualitative interview study. Br J Med Psychol. 1999;72(Pt 4):505-24.

13. Dura-Vila G, Hagger M, Dein S, Leavey G. Ethnicity, religion and clinical practice: a qualitative study of beliefs and attitudes of psychiatrists in the United Kingdom. Mental Health, Religion & Culture. 2011;14(1):53-64.

14. Edge D, Rogers A. Dealing with it: Black Caribbean women's response to adversity and psychological distress associated with pregnancy, childbirth, and early motherhood. Soc Sci Med. 2005;61(1):15-25.

15. Edge D. 'We don't see Black women here': an exploration of the absence of Black Caribbean women from clinical and epidemiological data on perinatal depression in the UK. Midwifery. 2008;24(4):379-89.

16. Edge D, MacKian SC. Ethnicity and mental health encounters in primary care: help-seeking and help-giving for perinatal depression among Black Caribbean women in the UK. Ethn Health. 2010;15(1):93-111.

17. Edge D. Falling through the net - black and minority ethnic women and perinatal mental healthcare: health professionals' views. Gen Hosp Psychiatry. 2010;32(1):17-25.

18. Faulkner A. Ethnic inequalities in mental health: Promoting lasting positive change: A consultation with black and minority ethnic mental health service users. In: Network NSU, editor. London: Lankelly Chase Foundation; 2014.

19. Fish M, Fakoussa O. Towards culturally inclusive mental health: learning from focus groups with those with refugee and asylum seeker status in Plymouth. International Journal of Migration Health and Social Care. 2018;14(4):361-76.

20. Green G, Bradby H, Chan A, Lee M, Eldridge K. Is the English National Health Service meeting the needs of mentally distressed Chinese women? J Health Serv Res Policy. 2002;7(4):216-21.

21. Green G, Bradby H, Chan A, Lee M. “We are not completely Westernised”: dual medical systems and pathways to health care among Chinese migrant women in England. Soc Sci Med. 2006;62(6):1498-509.

22. Gunputh V. An exploration of help-seeking among South Asians living in the UK: Canterbury Christ Church University; 2015.

23. Gurpinar-Morgan A, Murray C, Beck A. Ethnicity and the therapeutic relationship: views of young people accessing cognitive behavioural therapy. Mental Health, Religion & Culture. 2014;17(7):714-25.

24. Hills D, Aram E, Hinds D. Traditional healers action research project : final report prepared by The Tavistock Institute of Human Relations for The King's Fund. London: The Tavistock Institute of Human Relations, 2013.

25. Hussain FA, Cochrane R. Depression in South Asian women: Asian women's beliefs on causes and cures. Mental Health, Religion & Culture. 2002;5(3):285-311.

26. Jim J, Pistrang N. Culture and the therapeutic relationship: Perspectives from Chinese clients. Psychotherapy Research. Vol.17(4), 2007, pp. 461-473.

27. Kai J, Hedges C. Minority ethnic community participation in needs assessment and service development in primary care: perceptions of Pakistani and Bangladeshi people about psychological distress. Health Expect. 1999;2(1):7-20.

28. Kalathil J, Collier B, Bhakta R. Recovery and resilience: African, African-Caribbean and South Asian women's narratives of recovering from mental distress. London: Mental Health Foundation and Survivor Research, 2011.

29. Kang KK, Moran N. Experiences of inpatient staff meeting the religious and cultural needs of BAME informal patients and patients detained under the Mental Health Act 1983. Mental Health Review Journal. 2020.

30. Keating F, Robertson D, McCulloch A, Francis E. Breaking the circles of fear: A review of the relationship between mental health services and African and Caribbean communities. London: The Sainsbury Centre for Mental Health. 2002.

31. Keating F, Robertson D. Fear, black people and mental illness: a vicious circle? Health & social care in the community. 2004;12(5):439-47.

32. Klineberg E, Kelly MJ, Stansfeld SA, Bhui KS. How do adolescents talk about self-harm: a qualitative study of disclosure in an ethnically diverse urban population in England. BMC Public Health. 2013;13:572.

33. Li S, Hatzidimitriadou E, Psoinos M. "Tangled wires in the head": older migrant Chinese's perception of mental illness in Britain. J Aging Stud. 2014;30:73-86.

34. Mahmood ZJ. A qualitative exploration into how UK Pakistani male immigrants deal with personal problems and stresses in everyday life: London Metropolitan University; 2012.

35. Majumder P, O’Reilly M, Karim K, Vostanis P. ‘This doctor, I not trust him, I’m not safe’: The perceptions of mental health and services by unaccompanied refugee adolescents. Int J Soc Psychiatry. 2015;61(2):129-36.

36. Majumder P, Vostanis P, Karim K, O'Reilly M. Potential barriers in the therapeutic relationship in unaccompanied refugee minors in mental health. Journal of Mental Health. 2019;28(4):372-8.

37. Majumder P. Exploring stigma and its effect on access to mental health services in unaccompanied refugee children. Bjpsych Bulletin. 2019:275-81.

38. Mallinson S, Popay J. Describing depression: ethnicity and the use of somatic imagery in accounts of mental distress. Sociol Health Illn. 2007;29(6):857-71.

39. Mantovani N, Pizzolati M, Edge D. Exploring the relationship between stigma and help-seeking for mental illness in African-descended faith communities in the UK. Health Expect. 2017;20(3):373-84.

40. Marshall H, Yazdani A. Locating culture in accounting for self‐harm amongst Asian young women. J Community Appl Soc Psychol. 1999;9(6):413-33.

41. McEvoy P, Williamson T, Kada R, Frazer D, Dhliwayo C, Gask L. Improving access to mental health care in an Orthodox Jewish community: a critical reflection upon the accommodation of otherness. BMC Health Serv Res. 2017;17(1):1-15.

42. Mclean C, Campbell C, Cornish F. African-Caribbean interactions with mental health services in the UK: experiences and expectations of exclusion as (re) productive of health inequalities. Soc Sci Med. 2003;56(3):657-69.

43. Bhui K, Fenton S, Grewal I, Karlsen S, Lloyd K, Nazroo J, et al. Ethnic differences in context and experience of psychiatric illness : a qualitative study. London: National Centre for Social Research (NatCen), 2002.

44. Palmer D, Ward K. 'Lost': listening to the voices and mental health needs of forced migrants in London. Med Confl Surviv. 2007;23(3):198-212.

45. Patel N. How do people from the Indian Gujarati community make sense of help-seeking for mental health problems: Dissertation Abstracts International Section C: Worldwide. Vol.75(1-C),2018.

46. Rabiee F, Smith P. Being understood, being respected: An evaluation of mental health service provision from service providers and users' perspectives in Birmingham, UK. [References]: International Journal of Mental Health Promotion. Vol.15(3), 2013, pp. 162-177.

47. Rae S. Somali male refugees: Perceptions of depression and help-seeking: University of East London; 2014.

48. Reavey P, Ahmed B, Majumdar A. 'How can we help when she won't tell us what's wrong?' Professionals working with South Asian women who have experienced sexual abuse. [References]: Journal of Community & Applied Social Psychology. Vol.16(3), 2006, pp. 171-188.

49. Robinson M, Keating F, Robertson S. Ethnicity, gender and mental health. Diversity in Health & Care. 2011;8(2).

50. Selkirk M, Quayle E, Rothwell N. Influences on Polish migrants' responses to distress and decisions about whether to seek psychological help. Counselling Psychology Review. Vol.27(3), 2012, pp. 40-54.

51. Shefer G, Rose D, Nellums L, Thornicroft G, Henderson C, Evans-Lacko S. ‘Our community is the worst’: The influence of cultural beliefs on stigma, relationships with family and help-seeking in three ethnic communities in London. Int J Soc Psychiatry. 2013;59(6):535-44.

52. Tuffour I, Simpson A, Reynolds L. Mental illness and recovery: An interpretative phenomenological analysis of the experiences of Black African service users in England. J Res Nurs. 2019;24(1-2):104-18.

53. Upadhya B. How do young Nepalese people living in the UK make sense of mental health and problems of mental health: A qualitative exploration: Dissertation Abstracts International Section C: Worldwide. Vol.74(12-C), 2015.

54. Vincent F, Jenkins H, Larkin M, Clohessy S. Asylum-seekers' experiences of trauma-focused cognitive behaviour therapy for post-traumatic stress disorder: a qualitative study. Behav Cogn Psychother. 2013;41(5):579-93.

55. Wagstaff C, Graham H, Farrell D, Larkin M, Nettle M. Experiences of mental health services for 'black' men with schizophrenia and a history of disengagement: A qualitative study. Int J Ment Health Nurs. 2018;27(1):158-67.

56. Weatherhead S, Daiches A. Muslim views on mental health and psychotherapy. Psychology & Psychotherapy: Theory, Research & Practice. 2010;83(Pt 1):75-89.

57. Yeung EYW, Irvine F, Ng SM, Tsang KMS. How people from Chinese backgrounds make sense of and respond to the experiences of mental distress: Thematic analysis. J Psychiatr Ment Health Nurs. 2017;24(8):589-99.

58. Bailey NV, Tribe R. A qualitative study to explore the help-seeking views relating to depression among older Black Caribbean adults living in the UK. Int Rev Psychiatry. 2021;33(1-2):113-8.

59. Hussain B, Sheikh AZ, Repper J, Stickley T, Timmons S, Shah MH. Recognizing service users' diversity: social identity narratives of British Pakistanis in a mental health context. Journal of Mental Health Training, Education & Practice. 2021;16(3):200-12.

60. Lawrence V, McCombie C, Nikolakopoulos G, Morgan C. Ethnicity and power in the mental health system: experiences of white British and black Caribbean people with psychosis. Epidemiol Psychiatr Sci. 2021;30:e12.

61. Meechan H, John M, Hanna P. Understandings of mental health and support for Black male adolescents living in the UK. Children and Youth Services Review. 2021;129:106192.

62. Olaniyan F-V, Hayes G. Just ethnic matching? Racial and ethnic minority students and culturally appropriate mental health provision at British universities. International Journal of Qualitative Studies on Health and Well being. 2022;17(1):2117444.

63. Pilav S, De Backer K, Easter A, Silverio SA, Sundaresh S, Roberts S, et al. A qualitative study of minority ethnic women's experiences of access to and engagement with perinatal mental health care. BMC Pregnancy Childbirth. 2022;22(1):1-13.

64. Dare O, Jidong DE, Premkumar P. Conceptualising mental illness among University students of African, Caribbean and similar ethnic heritage in the United Kingdom. Ethn Health. 2022:1-22.

65. Lawrence V, McCombie C, Nikolakopoulos G, Morgan C. Navigating the mental health system: Narratives of identity and recovery among people with psychosis across ethnic groups. Soc Sci Med. 2021;279:113981.

66. Simkhada B, Vahdaninia M, van Teijlingen E, Blunt H. Cultural issues on accessing mental health services in Nepali and Iranian migrants communities in the UK. Int J Ment Health Nurs. 2021;30(6):1610-9.
